# Glucosinolate Induction and Resistance to the Cabbage Moth, *Mamestra brassicae*, Differs among Kale Genotypes with High and Low Content of Sinigrin and Glucobrassicin

**DOI:** 10.3390/plants10091951

**Published:** 2021-09-18

**Authors:** Francisco Rubén Badenes-Pérez, María Elena Cartea

**Affiliations:** 1 Instituto de Ciencias Agrarias, Consejo Superior de Investigaciones Científicas, 28006 Madrid, Spain; 2 Misión Biológica de Galicia, Consejo Superior de Investigaciones Científicas, 36080 Pontevedra, Spain; ecartea@mbg.csic.es

**Keywords:** *Brassica oleracea* var. *acephala*, glucosinolates, herbivory, host-plant resistance, jasmonic acid, salicylic acid

## Abstract

The cabbage moth, *Mamestra brassicae* L. (Lepidoptera: Noctuidae), is a generalist insect pest of cruciferous crops. We tested glucosinolate induction by jasmonic acid (JA) and salicylic acid (SA), and by these phytohormones combined with feeding by *M. brassicae* larvae in four genotypes of kale, *Brassica oleracea* L. var. *acephala* (Brassicaceae). The genotypes tested had high glucobrassicin (genotype HGBS), low glucobrassicin (genotype LGBS), high sinigrin (genotype HSIN), and low sinigrin content (genotype LSIN). Application of JA increased indolic and total glucosinolate content in all kale genotypes 1, 3, and 9 days after treatment. For SA-treated plants, glucosinolate induction varied depending on the number of days after treatment and the genotype. Overall, herbivory by *M. brassicae* accentuated and attenuated the effects of JA and SA, respectively, on plant glucosinolate content. Larvae of *M. brassicae* gained less weight on leaves from plants treated with JA compared to leaves from control plants and plants treated with SA. In bioassays with leaf discs, a significant reduction of defoliation only occurred in JA-treated plants of the HSIN genotype. This research shows that previous herbivory alters the susceptibility of kale to *M. brassicae* and that induction of glucosinolates varies among kale genotypes differing in their glucosinolate content.

## 1. Introduction

Plants in the family Brassicaceae contain glucosinolates that can be used for plant defense [1,2]. Unlike insects, specialists that are well adapted and even favored by glucosinolates in their host-plants, generalists are usually negatively affected by glucosinolate content [3,4,5,6,7,8]. This is the case for the cabbage moth, *Mamestra brassicae* L. (Lepidoptera: Noctuidae), for which high concentrations of glucosinolates have a detrimental effect on larval growth and survival [9,10,11,12]. For the development of larvae of this generalist, aliphatic glucosinolates have been shown to be more detrimental than indolic glucosinolates [13]. The aliphatic glucosinolates gluconapin, glucoiberin, and sinigrin have been associated to reduced performance of *M. brassicae* on different populations of *Brassica oleracea* L. (Brassicaceae) [14,15]. Experiments with different genotypes of kale, *B. oleracea* L. var. *acephala* (Brassicaceae), differing in the content of the aliphatic glucosinolates sinigrin and glucoiberin, and the indolic glucosinolate glucobrassicin, indicated that high content of these glucosinolates negatively affected larval weight in *M. brassicae* [11].

Jasmonic acid (JA) and salicylic acid (SA) modulate plant defense against different herbivores [16,17,18]. JA induces resistance against chewing herbivores, while phloem-feeders, which produce less injury to plant foliage, are perceived as pathogens and activate the SA signaling pathway [19]. Thus, JA and SA application can be used to simulate herbivory. Previous herbivory can affect plant responses to subsequent herbivory [20]. For example, in *B. oleracea*, previous attack by the phloem-feeder aphid *Brevicoryne brassicae* L. (Hemiptera: Aphididae) facilitated the herbivory in the chewing larvae of *Pieris rapae* L. (Lepidoptera: Pieridae), which developed faster and gained more weight on plants previous infested by *B. brassicae* [20].

JA and SA can affect induction of glucosinolates differently [21,22]. In *Brassica rapa* L. (Brassicaceae), SA application caused a greater increase in the aliphatic and aromatic glucosinolates sinigrin and gluconasturtiin, respectively, but a lesser increase in the indolic glucosinolates glucobrassicin and 4-methoxyglucobrassicin compared to JA application [23]. Feeding by lepidopteran larvae can induce glucosinolate content in plants [4,24,25]. In *Brassica napus* L. (Brassicaceae), feeding by *M. brassicae* larvae increased levels of the indolic glucosinolates glucobrassicin and neoglucobrassicin [26]. Thus, we expect that application of JA and SA will differently affect plant glucosinolate content and herbivory by *M. brassicae*. In the case of a chewing herbivore like *M. brassicae* larvae, JA application should have a more detrimental effect than SA application.

The objectives of this research were to compare glucosinolate induction, herbivory, and larval growth of *M. brassicae* after JA and SA application in four different plant genotypes selected for high and low glucobrassicin and sinigrin content. We also studied glucosinolate induction under the combination of either JA or SA application and feeding by *M. brassicae* larvae. In plants previously treated by these phytohormones, herbivory by *M. brassicae* is likely to boost and offset the effects of JA of SA, respectively. After treatment, we compared how glucosinolate changed through time 1, 3, and 9 days after treatment. We also compared the effect of each treatment on the glucosinolate content in each genotype at each time of analysis, and the differences in glucosinolate content among genotypes for each treatment and time of analysis. In the latter case, besides the actual glucosinolate content, we also looked at glucosinolate changes as a percentage of variation compared to the control within each genotype.

## 2. Results

### 2.1. Glucosinolate Content in Kale Genotypes

**Figure 1:**
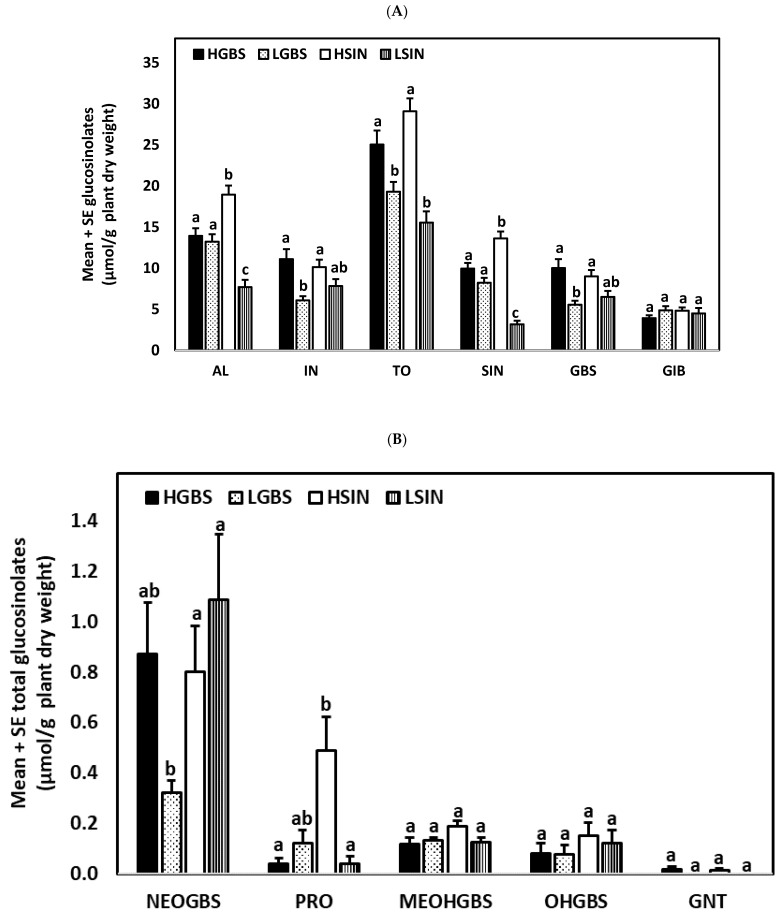
Mean ± SE glucosinolate content (µmol g^−1^ plant dry weight) in kale genotypes high in glucobrassicin (HGBS), low in glucobrassicin (LGBS), high in sinigrin (HSIN), and low in sinigrin (LSIN). The glucosinolates shown are total aliphatic (AL), total indolic (IN), total glucosinolates (TO), sinigrin (SIN), glucobrassicin (GBS), and glucoiberin (GIB) (**A**), and neoglucobrassicin (NEO), progoitrin (PRO), 4-methoxyglucobrassicin (MEOHGBS), 4-hydroxyglucobrassicin (OHGBS), and gluconasturtiin (GNT) (**B**). Post hoc tests with a significance level of *p* ≤ 0.05 were run to compare differences in glucosinolate content among genotypes. Significant differences are shown with different lowercase letters.

Analyzed over the length of the study, i.e., all control plants in each of the four kale genotypes combining the three time points, plants of the different genotypes differed in total aliphatic (AL), total indolic (IN), and total glucosinolate content (TO) (*p* ≤ 0.001), as well as in sinigrin (SIN) (*p* ≤ 0.001), glucobrassicin (GBS) (*p* ≤ 0.001), neoglucobrassicin (NEO) (*p* = 0.036), and progoitrin (PRO) (*p* ≤ 0.001) (Figure 1). Differences in the content of glucoiberin (GIB) (*p* = 0.540), 4-hydroxyglucobrassicin (OHGBS) (*p* = 0.612), 4-methoxyglucobrassicin (MEOHGBS) (*p* = 0.075), and gluconasturtiin (GNT) (*p* = 0.212) were not statistically significant. AL content was significantly higher in the HSIN genotype and significantly lower in LSIN than in the other genotypes. IN content was significantly higher in the HGBS and HSIN genotypes than in the LGBS genotype. TO content was higher in the genotypes HGBS and HSIN than in the genotypes LGBS and LSIN. Among individual glucosinolates, SIN content was higher in the genotype HSIN and lower in the genotype LSIN than in the other genotypes (Figure 1). This is the reason why we refer to these genotypes as HSIN and LSIN. GBS content was significantly higher in the HGBS and HSIN genotypes than in the LGBS genotype (Figure 1). Because of this, we refer to the other two genotypes used in this study as HGBS and LGBS. PRO content was higher in the genotype HSIN than in the genotypes HGBS and LSIN. In the experiments that we describe hereafter, we will focus on the main glucosinolate groups AL, IN, and TO. The most abundant glucosinolates GIB, SIN, GBS, and NEO are shown in the text only when comparisons of glucosinolate content among treatments and genotypes are shown as percentage increases (Table 1) and also in tables as supplementary data. The other less abundant glucosinolates are only shown as supplementary data (Table S1).

### 2.2. Glucosinolate Induction over Time after Phytohormone and Herbivory Treatments

#### 2.2.1. Control Plants

**Figure 2:**
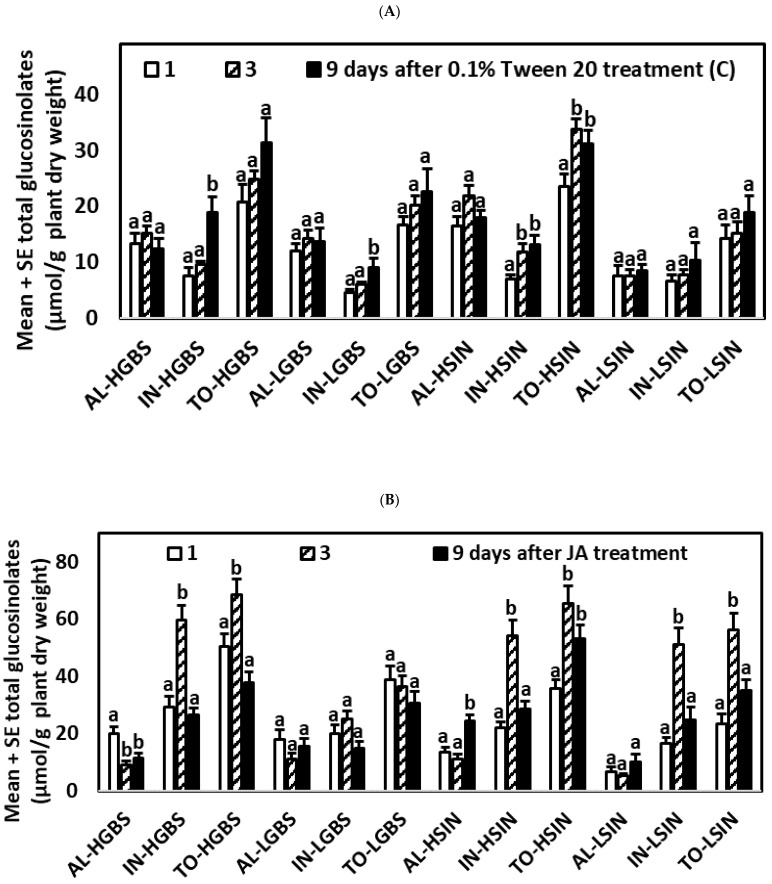

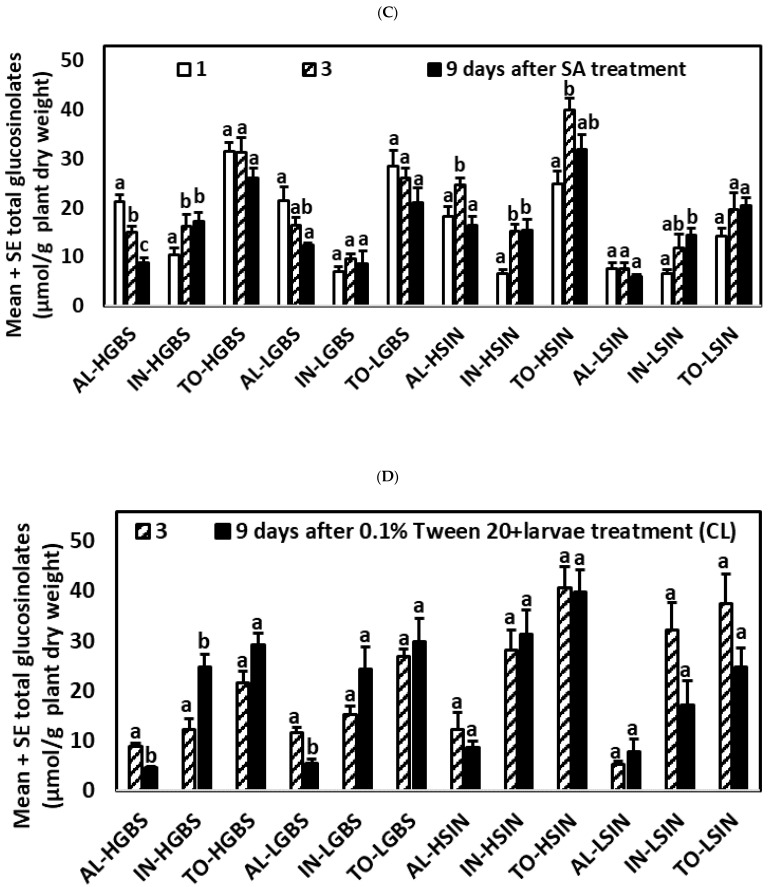

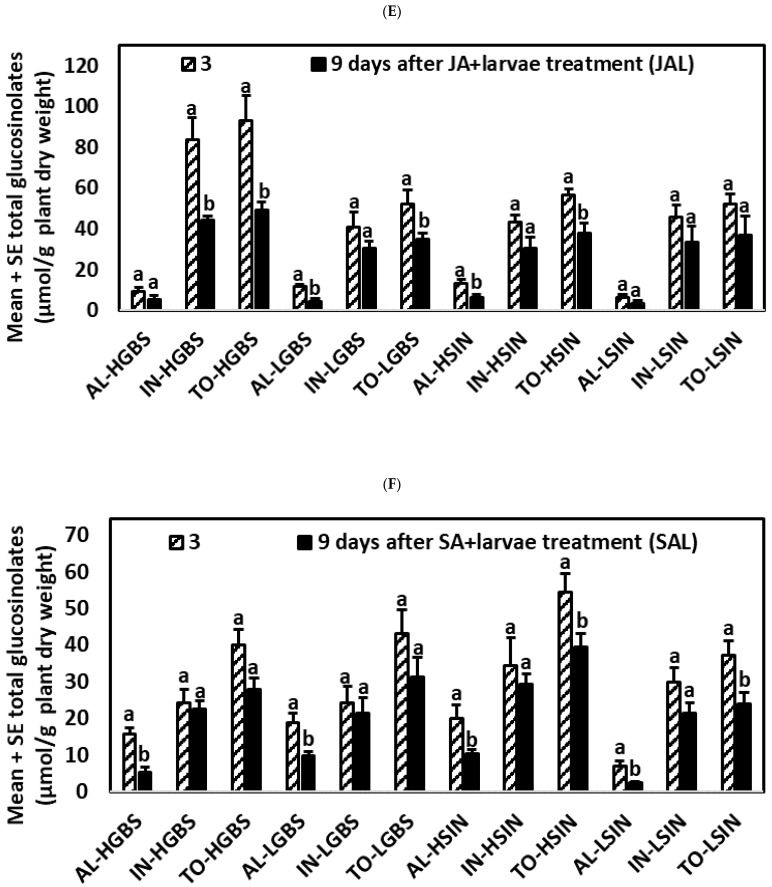
Mean ± SE glucosinolate content (µmol g^−1^ plant dry weight) in kale genotypes high in glucobrassicin (HGBS), low in glucobrassicin (LGBS), high in sinigrin (HSIN), and low in sinigrin (LSIN) 1, 3, and 9 days after treatment, respectively. The treatments are control (C) (**A**), jasmonic acid (JA) (**B**), salicylic acid (SA) (**C**), control with larvae (CL) (**D**), JA with larvae (JAL) (**E**), and SA with larvae (SAL) (**F**). The glucosinolates shown are total aliphatic (AL), total indolic (IN), and total glucosinolates (TO). Post hoc tests with a significance level of *p* ≤ 0.05 were run to compare differences among times after treatment within subgroups of total glucosinolates and genotype. Significant differences are shown with different lowercase letters.

For control plants of the HGBS, LGBS, and HSIN genotypes significant differences across times were found for IN (*p*-values in Table S2). IN contents were highest 9 days after treatments began (Table S3, Figure 2A). In plants of the HSIN genotype TO contents were higher after 3 and 9 days than after 1 day. For plants of the LSIN genotype no significant differences across times were found for the major glucosinolate groups. AL contents did not significantly change through the time of the experiment for any of the four genotypes.

#### 2.2.2. Plants Treated with JA

For plants of the HGBS and HSIN genotypes, significant differences across times were found for AL, IN, and TO (Tables S2 and S3; Figure 2B). Concentrations of AL were highest 1 and 9 days after treatment for plants of the HGBS and HSIN genotypes, respectively. For IN and TO, concentrations were highest 3 days after treatment. For plants of the LGBS genotype, differences across times were not significant for the main glucosinolates. For plants of the LSIN genotype, significant differences across times were found for IN and TO, which contents were highest after 3 days. Thus, for JA-treated plants of the HGBS, HSIN, and LSIN genotypes, IN and TO contents were highest 3 days after treatment.

#### 2.2.3. Plants Treated with SA

Significant differences across times for AL were found for all genotypes, except LSIN. Significant differences across times for IN were found for all genotypes, except LGBS. For plants of the HGBS genotype significant differences across times were found for AL and IN (Tables S2 and S3; Figure 2C). For plants of the LGBS genotype significant differences across times were found for AL, which contents were higher 1 day than 9 days after treatment. For plants of the HSIN genotype significant differences across times were found for AL, IN, and TO. For plants of the LSIN genotype significant differences across times were found for IN. SA-treated plants of the HGBS and LGBS genotypes showed the highest AL contents 1 day after treatment, while in plants of the HSIN genotype, AL contents were highest 3 days after treatment. SA-treated plants of the HGBS and LSIN genotypes had in common that IN were highest 9 days after treatment. However, in the case of the HSIN genotype, IN contents did not differ 3 and 9 days after treatment but were higher than 1 day after treatment.

#### 2.2.4. Control Plants with Larval Herbivory (CL Treatment)

For plants of the HGBS genotype, significant differences across times were found for AL and IN (Tables S2 and S3; Figure 2D). For this genotype, concentrations of AL were higher 3 days after the experiment began (2 days after larval feeding started) than 9 days after the experiment began (8 days after larval feeding started). The opposite was found for IN, which concentrations were lower 3 days than 9 days after the experiment began. For plants of the LGBS genotype, significant differences across times were found for AL, which concentrations were also higher 3 days than 9 days after the experiment began. Thus, for CL plants, in both HGBS and LGBS genotypes, AL contents were highest 3 days after the experiment began. For plants of the HSIN and LSIN genotypes, differences across times were not significant for the main glucosinolates.

#### 2.2.5. Plants Treated with JA and Larval Herbivory (JAL Treatment)

For plants of the HGBS genotype, significant differences across times were found for IN and TO (Tables S2 and S3, Figure 2E), which concentrations were highest 3 days after the experiment began. HGBS was the only genotype in which IN content was significantly induced after JAL treatment. JAL plants of the LGBS and HSIN genotypes both had AL and TO contents that were higher 3 days than 9 days after the experiment began. In plants of the HGBS genotype, TO contents were also higher 3 days than 9 days after the experiment began. For plants of the LSIN genotype, no significant differences across times were found for the main glucosinolate groups.

#### 2.2.6. Plants Treated with SA and Larval Herbivory (SAL Treatment)

For plants of the four genotypes, significant differences across times were found for AL (Tables S2 and S3; Figure 2F), which concentrations were higher 3 days than 9 days after the experiment began. This highest content 3 days after the experiment began also occurred for TO in the HSIN and LSIN genotypes.

### 2.3. Differences in Glucosinolate Induction among Treatments and Kale Genotypes: Effect of Phytohormone and Herbivory Treatments

#### 2.3.1. One Day after the Application of Phytohormones

**Figure 3:**
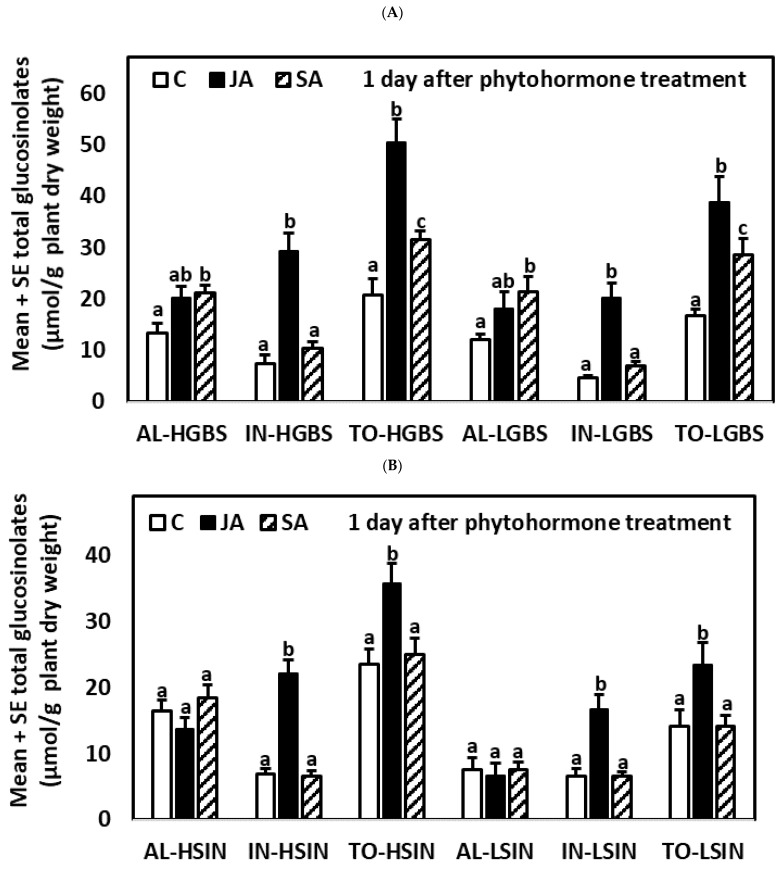
Mean ± SE glucosinolate content (µmol g^−1^ plant dry weight) in kale genotypes high in glucobrassicin (HGBS) and low in glucobrassicin (LGBS) (**A**), and high in sinigrin (HSIN) and low in sinigrin (LSIN) (**B**). Data shown are from plants one day after application of phytohormones. The treatments are jasmonic acid (JA), salicylic acid (SA), and control (C). The glucosinolates shown are total aliphatic (AL), total indolic (IN), and total glucosinolates (TO). Post hoc tests with a significance level of *p* ≤ 0.05 were run to compare differences among phytohormone treatments within subgroups of total glucosinolates and genotype. Significant differences are shown with different lowercase letters.

One day after the application of phytohormones, plants of all genotypes showed significant differences among treatments for IN and TO (*p*-values in Table S4) (Table S5, Figure 3). Plants of the HGBS and LGBS genotypes also showed significant differences between treatments for AL, but these differences were not significant in plants of the HSIN and LSIN genotypes. Plants treated with JA had higher content of IN and TO than plants in the control and SA treatments in the four genotypes tested. In plants of the HGBS and LGBS genotypes, AL and TO contents were higher in SA-treated than in control plants, but these differences were not significant in plants of the HSIN and LSIN genotypes.

#### 2.3.2. Three Days after the Application of Phytohormones

**Figure 4:**
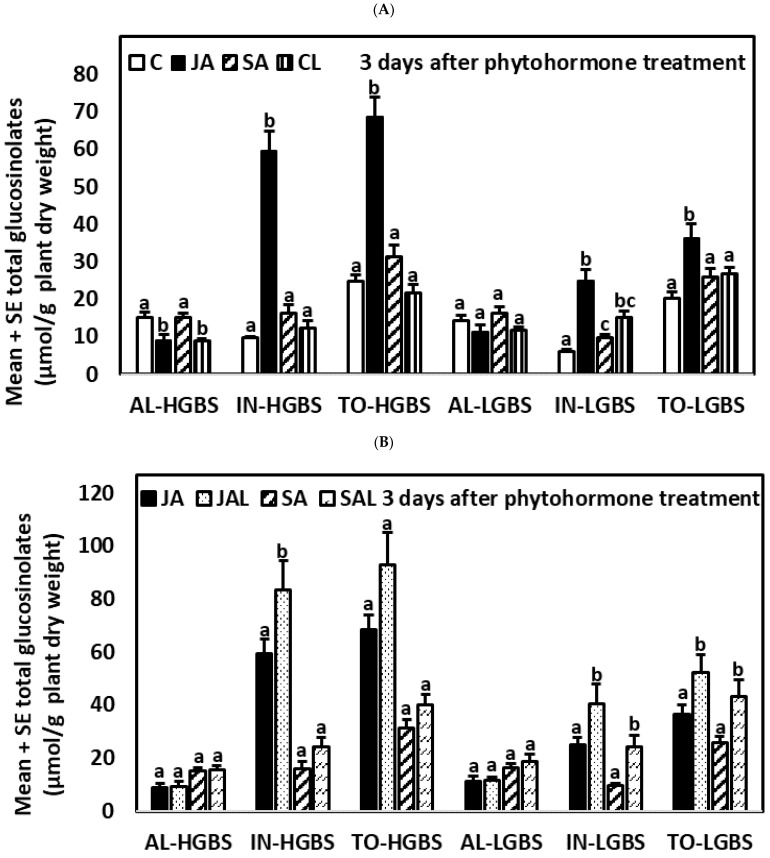

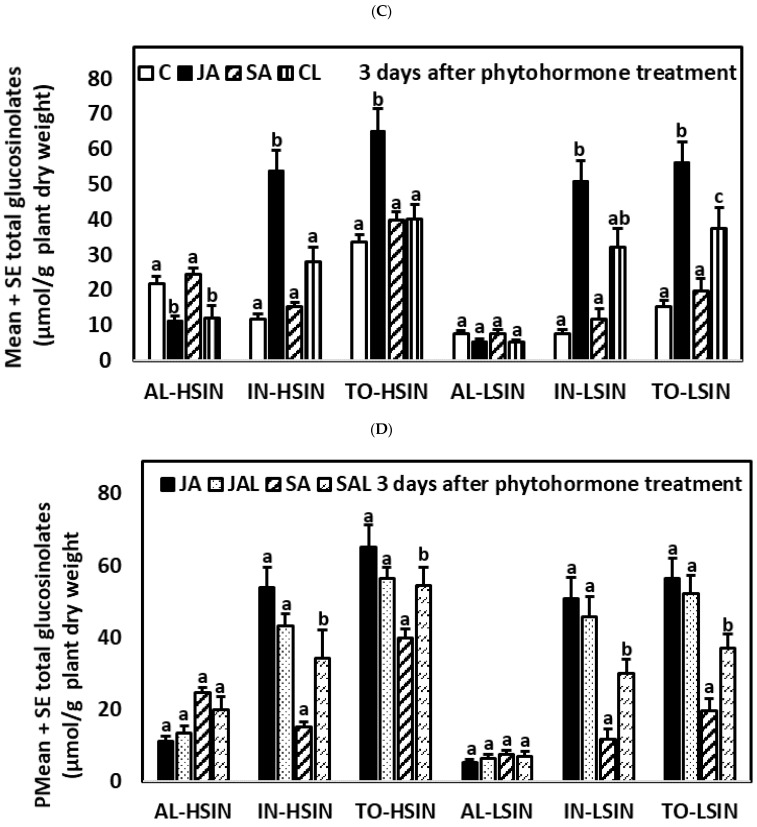
Mean ± SE glucosinolate content (µmol g^−1^ plant dry weight) in kale genotypes high in glucobrassicin (HGBS), low in glucobrassicin (LGBS) (**A**,**B**), high in sinigrin (HSIN), and low in sinigrin (LSIN) (**C**,**D**). Data shown are from plants three days after application of phytohormones. The treatments are jasmonic acid (JA), salicylic acid (SA), JA with larvae (JAL), SA with larvae (SAL), control (C), and control with larvae (CL). The glucosinolates shown are total aliphatic (AL), total indolic (IN), and total glucosinolates (TO). Post hoc tests with a significance level of *p* ≤ 0.05 were run to compare differences among phytohormone treatments within subgroups of total glucosinolates and genotype. Significant differences are shown with different lowercase letters.

Three days after the application of JA and SA, IN and TO contents continued to be higher in JA-treated plants than in plants treated with SA and control plants (Tables S4 and S5; Figure 4). JA treatment increased IN and decreased AL contents in plants of the high glucosinolate genotypes HGBS and HSIN. In contrast, the SA treatment had no significant effect on glucosinolates in plants of any of the genotypes.

Herbivory (CL treatment) had a clear effect on the variation of glucosinolates with respect to the control plants. It caused a decrease in AL content in the high glucosinolate genotypes (HSIN and HGBS), while plants in the LSIN genotype showed increased TO content. An increase in IN content was observed in the LGBS genotype. Compared to JA-treated plants, CL plants had similar levels of AL and lower content of TO in all genotypes. Thus, overall, induction of TO was greater in JA-treated plants than in plants with only larval herbivory. Compared to SA-treated plants, CL plants had lower content of AL in the high glucosinolate genotypes HGBS and HSIN, and higher content of TO in the LSIN genotype.

When comparing JAL and SAL plants (plants of the JA and SA treatments combined with *M. brassicae* larvae), differences in IN and TO content became non-significant as a result of larval feeding, except in the HGBS genotype, in which JAL plants had higher IN and TO content than SAL plants (Tables S4 and S6; Figure 4). Plants from the JAL treatment had higher IN content than JA-treated plants in the HGBS and LGBS genotypes selected for content of the indolic glucosinolate GBS, but these differences were not significant in the case of the HSIN and LSIN genotypes selected for the aliphatic glucosinolate SIN. Except for the HGBS genotype, in which differences were not significant, plants from the SAL treatment had higher contents of IN and TO than SA-treated plants. Therefore, overall, in plants previously treated by JA and SA, larval herbivory increased IN content 3 days after treatment, although this depended on the treatment and genotype.

#### 2.3.3. Nine Days after the Application of Phytohormones

**Figure 5:**
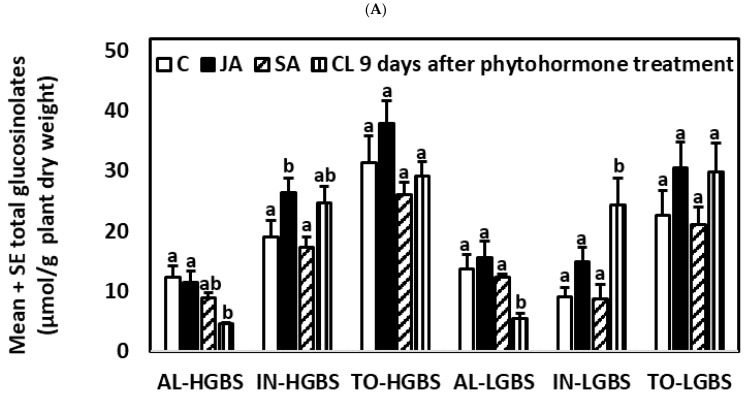

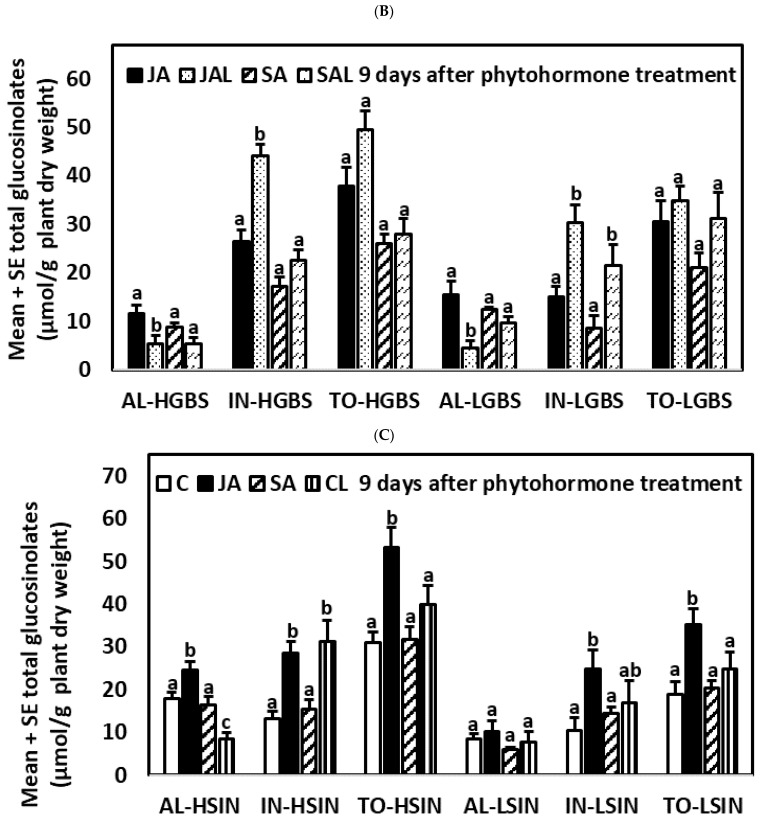

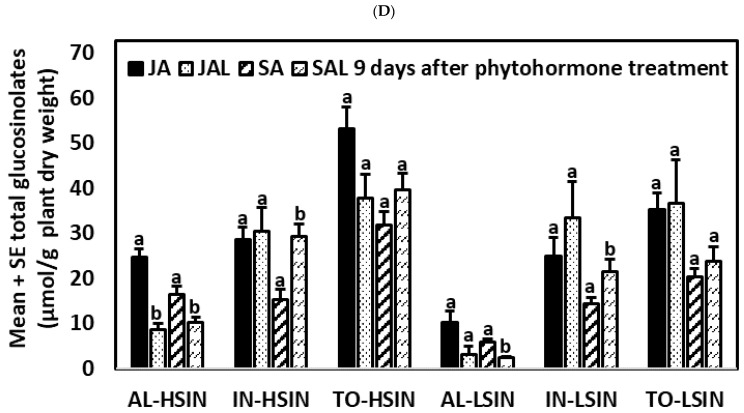
Mean ± SE glucosinolate content (µmol g^−1^ plant dry weight) in kale genotypes high in glucobrassicin (HGBS), low in glucobrassicin (LGBS) (**A**,**B**), high in sinigrin (HSIN), and low in sinigrin (LSIN) (**C**,**D**). Data shown are from plants nine days after application of phytohormones. The treatments are jasmonic acid (JA), salicylic acid (SA), JA with larvae (JAL), SA with larvae (SAL), control (C), and control with larvae (CL). The glucosinolates shown are total aliphatic (AL), total indolic (IN), and total glucosinolates (TO). Post hoc tests with a significance level of *p* ≤ 0.05 were run to compare differences among phytohormone treatments within subgroups of total glucosinolates and genotype. Significant differences are shown with different lowercase letters.

Nine days after the application of phytohormones, IN content continued to be higher in JA-treated plants than in control plants in all genotypes, except LGBS (Tables S4 and S5, Figure 5). JA-treated plants also had higher content of TO content than control plants in the HSIN and LSIN genotypes. JA-treated plants of the LSIN genotype also had higher content of AL than control plants. Differences in glucosinolate content between JA- and SA-treated plants and between control and SA-treated plants were no longer significant.

The CL treatment had lower content of AL than control and JA-treated plants without larvae, except in the LSIN genotype, in which these differences were not significant. Plants in the CL treatment also had lower content of AL than SA plants of the HSIN and LGBS genotypes, but not in the others. In the LGBS genotype, CL plants had higher content of IN than control plants, JA-treated, and SA-treated plants, but in the other genotypes differences between these treatments were not significant.

Plants from the JAL treatment had lower AL and higher IN contents than JA-treated plants of the HGBS and LGBS genotypes (Tables S4 and S6; Figure 5). Plants from the JAL treatment also had lower content of AL than JA-treated plants of the HSIN treatment. Plants from the SAL treatment had lower AL and higher IN contents than SA-treated plants of the HSIN and LSIN genotypes. Plants from the SAL treatment also had higher content of IN than SA-treated plants of the LGBS treatment. There were no significant differences in TO content between plants JA and JAL treatments nor between plants of the SA and SAL treatments. Therefore, overall, in plants previously treated with JA and SA, larval herbivory decreased and increased AL and IN contents, respectively, although this depended on the treatment and genotype.

### 2.4. Differences in Glucosinolate Induction among Treatments and Kale Genotypes: Glucosinolate Differences among Genotypes

#### 2.4.1. One Day after the Application of Phytohormones

In control plants, there were significant differences among genotypes for AL and TO (*p*-values in Table S7) (Table S8). AL and TO contents were higher in plants of the HSIN genotype than in plants of the LSIN genotype. TO content was also higher in plants of the HSIN genotype than in plants of the LGBS genotype.

In JA-treated plants, there were significant differences among genotypes for AL, IN, and TO, which contents were lowest in plants of the LSIN genotype. In SA-treated plants, there were significant differences among genotypes for AL, IN, and TO. The contents of AL and TO in SA-treated plants were lowest in plants of the LSIN genotype, while IN content was highest in the HGBS genotype. Thus, 1 day after the application of phytohormones, both JA- and SA-treated plants had in common that content of AL and TO were lowest in plants of the LSIN genotype. Compared to plants in the control treatment, in which there were no significant differences in IN content among genotypes, the application of JA and SA resulted in significant differences among genotypes for IN content.

#### 2.4.2. Three Days after the Application of Phytohormones

In control plants, there were significant differences among genotypes for AL, IN, and TO (Tables S7 and S8). AL and TO were highest in plants of the HSIN genotype, while IN contents were higher in plants of the HGBS and HSIN genotypes than in plants of the LGBS genotype.

In JA-treated plants, there were significant differences among genotypes for AL, IN, and TO. Contents of IN and TO were lowest in JA-treated plants of the LGBS genotype, while contents of AL were lowest in the LSIN genotype. In SA-treated plants, there were significant differences among genotypes for AL and TO, which were lowest in the LSIN genotype, but there were no significant differences among genotypes for IN. Thus, compared to plants of the control treatment, in SA-treated plants differences in IN content among genotypes were no longer significant. In the CL treatment, there were significant differences among genotypes for IN and TO, the contents of which were higher in plants of the HSIN and LSIN genotypes than in plants of the HGBS genotype. Content of TO was also higher in CL-treated plants of the HSIN genotype than in CL-treated plants of the LGBS genotype. Compared to the control plants, as a result of larval feeding, IN contents were no longer higher in plants of the genotype HGBS than in plants of the genotype LGBS.

In the JAL treatment, there were significant differences among genotypes for IN and TO. IN and TO contents were highest in the HGBS genotype. In the SAL treatment, there were significant differences among genotypes for AL, which contents were lowest in the LSIN genotype. Compared to the control plants, in JAL- and SAL-treated plants IN contents were no longer higher in plants of the genotype HGBS than in plants of the genotype LGBS. Plants in the JA, SA, and SAL treatments had in common that content of AL continued to be lowest in plants of the LSIN genotype 3 days after the application of phytohormones.

#### 2.4.3. Nine Days after the Application of Phytohormones

In control plants, there were significant differences among genotypes for AL and IN (Tables S7 and S8). AL contents were higher in plants of the HSIN genotype than in plants of the LSIN and HGBS genotypes, while IN contents were higher in plants of the HGBS genotype than in plants of the LSIN and LGBS genotypes.

In both JA- and SA-treated plants, there were significant differences among genotypes for AL, IN, and TO; contents of AL and TO were highest in the HSIN genotype, while content of IN was lowest in the LGBS genotype. Regarding the effect of induction by larval feeding, in plants of the CL and JAL treatments, there were no significant differences among genotypes in the contents of the main glucosinolate groups. In the SAL treatment, there were significant differences among genotypes for AL, which contents were higher in plants of the LGBS and HSIN genotypes than in plants of the HGBS and LSIN genotypes. Compared to the control plants, in CL-, JAL-, and SAL-treated plants, IN contents were no longer higher in plants of the genotype HGBS than in plants of the genotype LGBS; in the case of the CL- and JAL- treated plants, AL contents were no longer higher in plants of the HSIN genotype than in plants of the LSIN and HGBS genotypes.

### 2.5. Differences in Glucosinolate Induction among Treatments and Kale Genotypes: Percent Glucosinolate Variation among Genotypes

#### 2.5.1. One Day after the Application of Phytohormones

**Figure 6:**
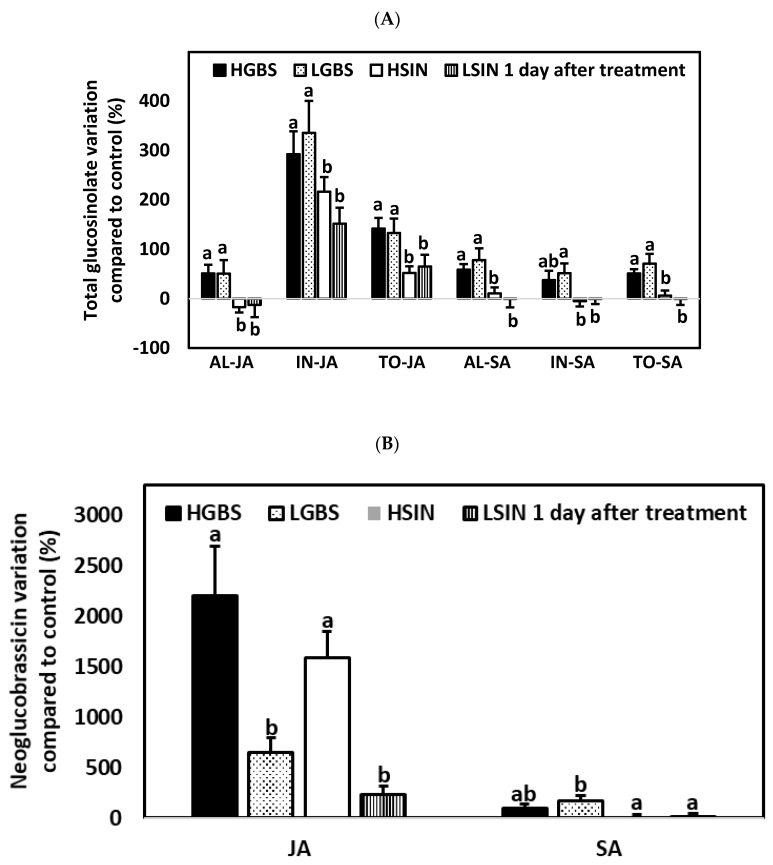
Mean ± SE percentage glucosinolate content related to control plants in kale genotypes high in glucobrassicin (HGBS), low in glucobrassicin (LGBS), high in sinigrin (HSIN), and low in sinigrin (LSIN) one day after treatment with jasmonic acid (JA) and salicylic acid (SA). The glucosinolates shown are total aliphatic (AL), total indolic (IN), and total glucosinolates (TO) (**A**) and neoglucobrassicin (**B**). Post hoc tests with a significance level of *p* ≤ 0.05 were run to compare differences among genotypes within subgroups of treatment and glucosinolate content. Significant differences are shown with different lowercase letters.

Regarding differences in percent changes in glucosinolate content among genotypes compared to the controls of each genotype, one day after the application of phytohormones there were significant differences among genotypes for AL, IN, TO, GIB, and NEO in both JA and SA treatments (*p*-values in Table S9) (Table 1; Figure 6). In the JA treatment there were also significant differences among genotypes for SIN.

Percent changes in AL and TO contents in JA- and SA-treated plants were higher in plants of the HGBS and LGBS genotypes than in the HSIN and LSIN genotypes (Figure 6A). Changes in glucosinolate content among JA-treated plants of the four plant genotypes were mostly due to an increase in IN contents. In JA-treated plants, IN contents increased between 151.9% and 335.7%, and the percentage of increase was higher in plants of the genotypes HGBS and LGBS than in plants of the genotype LSIN. The glucosinolate that increased most was NEO, which increased more in the HGBS and HSIN genotypes (2198.0% and 1584.8% increases, respectively) than in the LGBS and LSIN genotypes (Figure 6B). In SA-treated plants, changes in IN contents ranged from a 5% decrease in plants of the HSIN genotype to a 51.6% increase in plants of the LGBS genotype. This percent change in IN content was higher in the genotype LGBS than in the genotypes HSIN and LSIN. As in the case of JA-treated plants, SA-treated plants showed an increase in NEO content, but this increase was not as high as in JA-treated plants. In SA-treated plants, NEO also showed a higher increase in the HGBS and LGBS genotypes (94.5% and 171.8% increases, respectively) than in the LSIN and HSIN genotypes. As a result of JA treatment, the glucosinolate GIB increased more in plants of the genotypes HGBS, BGBS, and HSIN (69.3%, 60.8%, and 24.2% increases, respectively) than in plants of the genotype LSIN, in which this glucosinolate decreased. Changes in glucosinolate content among SA-treated plants in the four plant genotypes were mostly due to the increase in GIB, which increased more in plants of the HGBS and LGBS genotypes (97.2% and 82.6% increases, respectively) than in plants of the HSIN and LSIN genotypes.

#### 2.5.2. Three Days after the Application of Phytohormones

**Figure 7:**
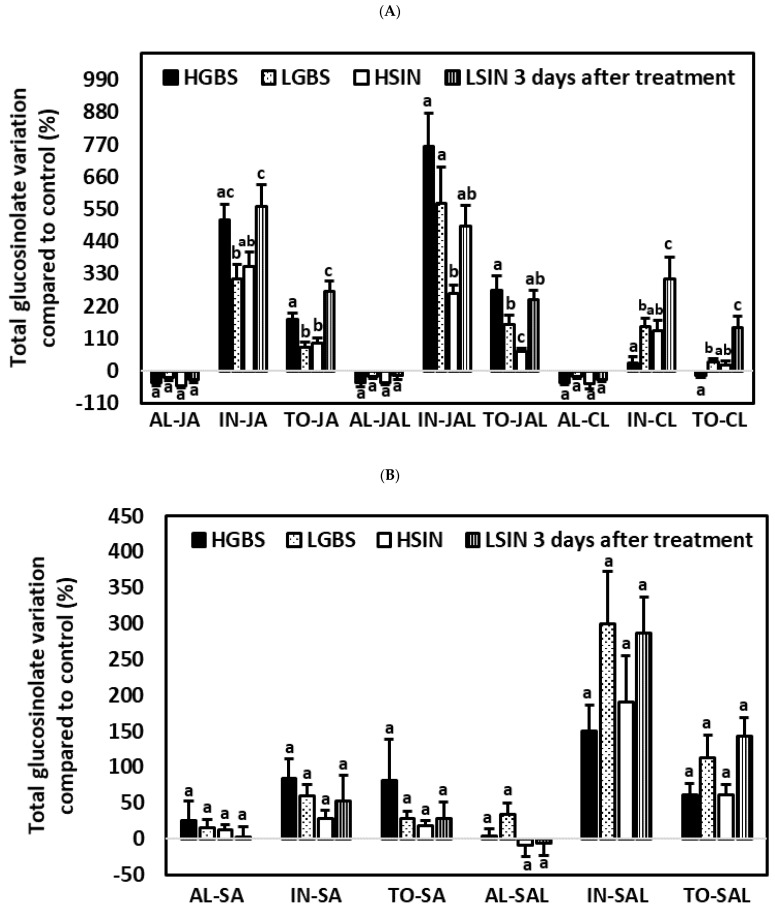

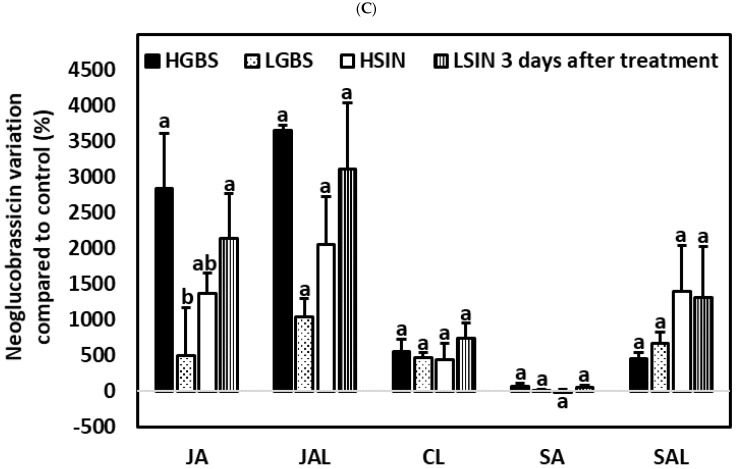
Mean ± SE percentage glucosinolate content related to control plants in kale genotypes high in glucobrassicin (HGBS), low in glucobrassicin (LGBS), high in sinigrin (HSIN), and low in sinigrin (LSIN). Data shown are from plants three days after application of phytohormones. The glucosinolates shown are total aliphatic (AL), total indolic (IN), and total glucosinolates (TO) for the treatments with jasmonic acid (JA), JA with larvae (JAL), and control with larvae (CL) (**A**) and after treatment with salicylic acid (SA), and SA with larvae (SAL) (**B**). Additionally, neoglucobrassicin is also shown for the treatments JA, JAL, CL, SA, and SAL (**C**). Post hoc tests with a significance level of *p* ≤ 0.05 were run to compare differences among genotypes within subgroups of treatment and glucosinolate content. Significant differences are shown with different lowercase letters.

There were no significant percent changes in AL content among the four genotypes in any of the treatments (Table S9). Percent changes in AL content were negative for all genotypes under the JA, JAL, and CL treatments and in the genotypes HSIN and LSIN under the SAL treatment. There were significant differences among genotypes for IN, TO, and NEO in the JA treatment (Table 1 and Table S9, Figure 7). In both the SA and SAL treatments, there were no significant differences among genotypes for any of the main glucosinolates. In the CL and JAL treatments there were significant differences among genotypes for IN, TO, and GBS.

In JA-treated plants percent changes in IN and TO content were highest in the HGBS and LSIN genotypes. In JA-treated plants IN contents increased between 313.6% in plants of the LGBS genotype and 335.7% in plants of the LSIN genotype. IN induction was mostly due to NEO increase. Percent increases in NEO contents in JA-treated plants were significantly higher in the HGBS and LSIN genotypes than in the LGBS genotype (Table 1 and Table S9; Figure 7C). The highest percent increase in NEO content occurred in the HGBS genotype (2837.5%). The percent increases in GBS in the HGBS genotype (355.3%) and in the LGBS genotype (300.4%) were not significantly different.

In plants of the JAL treatment, percent increase in TO contents was lower in the HSIN genotype than in the other genotypes. The percentage increase in IN was higher in the genotypes HGBS and LGBS (763.2% and 570.8%, respectively) than in the genotype HSIN. It was mostly due to NEO variation, ranging from 1039.6% in plants of the LGBS genotype to 3655.5% in plants of the HGBS genotype, but differences among genotypes were not significant. Percent increases in GBS were highest in plants of the HGBS and LGBS genotypes (568.9% and 545.6%, respectively).

In SA- and SAL-treated plants there were no significant differences in percent changes in AL, IN, TO, GIB, SIN, GBS, and NEO contents among the different plant genotypes.

In the CL treatment, percent changes in IN and TO content were higher in plants of the LSIN genotype than in the other genotypes. Among individual glucosinolates, the percent variation in GBS was highest in plants of the LSIN genotype (268.7%) and lowest in plants of the HGBS genotype, which showed a decrease of −11.6%.

#### 2.5.3. Nine Days after the Application of Phytohormones

**Figure 8:**
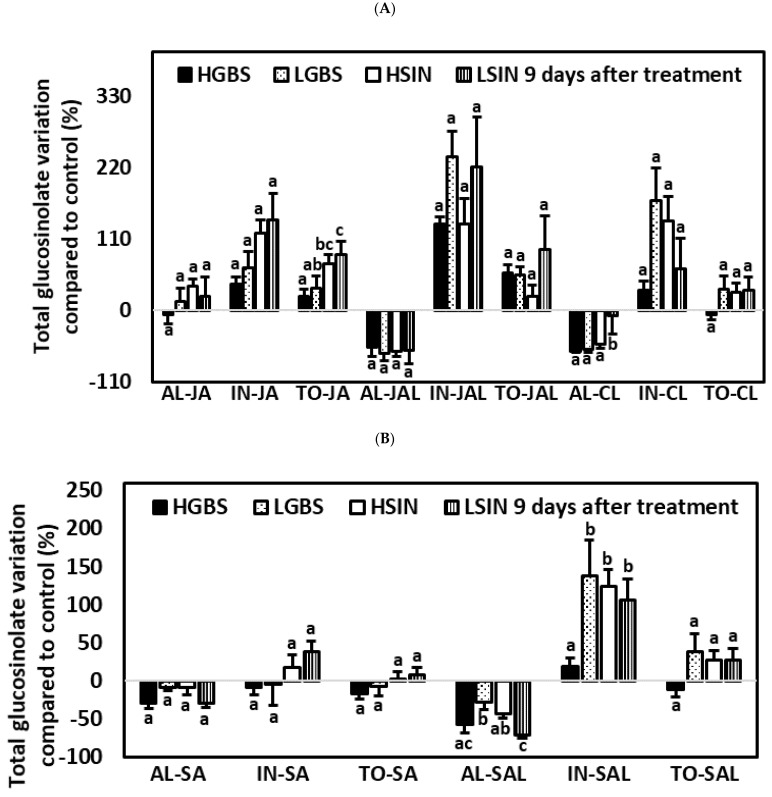

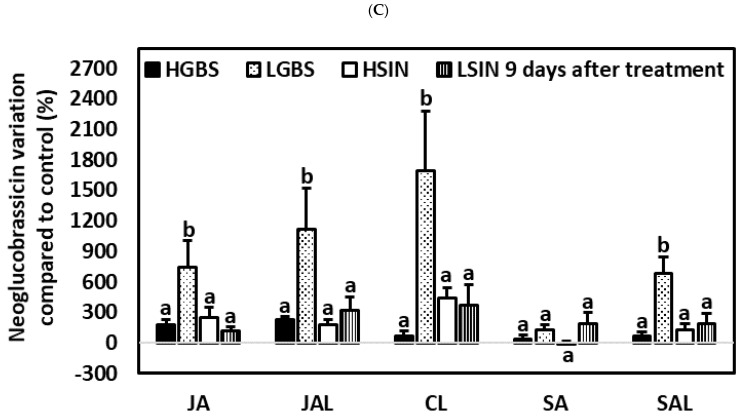
Mean ± SE percentage glucosinolate content related to control plants in kale genotypes high in glucobrassicin (HGBS), low in glucobrassicin (LGBS), high in sinigrin (HSIN), and low in sinigrin (LSIN). Data shown are from plants nine days after application of phytohormones. The glucosinolates shown are total aliphatic (AL), total indolic (IN), and total glucosinolates (TO) for the treatments with jasmonic acid (JA), JA with larvae (JAL), and control with larvae (CL) (**A**) and after treatment with salicylic acid (SA), and SA with larvae (SAL) (**B**). Additionally, neoglucobrassicin is also shown for the treatments JA, JAL, CL, SA, and SAL (**C**). Post hoc tests with a significance level of *p* ≤ 0.05 were run to compare differences among genotypes within subgroups of treatment and glucosinolate content. Significant differences are shown with different lowercase letters.

There were significant differences among genotypes for TO and NEO in the JA treatment, while in the SA treatment there were only significant differences among genotypes for SIN (Table 1 and Table S9, Figure 8). In the CL treatment, there were significant differences among genotypes for AL, GIB, and NEO. In the JAL treatment, there were only significant differences among genotypes for NEO. In the SAL treatment, there were significant differences among genotypes for AL, IN, GIB, SIN, and NEO.

Significant percent changes in TO content among plant genotypes occurred only in JA-treated plants, in which the percentage increase in TO content was highest in the HSIN and LSIN genotypes (71.4% and 86.7%, respectively).

Except in the SAL treatment, there were no significant differences among genotypes in percentage variation of IN, which percent increase was highest in plants of the LGBS and HSIN genotypes (138.2% and 123.9%, respectively).

Significant percentage changes in AL content occurred only in the CL and SAL treatments. In the case of the CL treatment, the percentage decrease in AL was not as much in plants of the LSIN genotype (−7.8%) as in plants of the other genotypes. However, in the case of the SAL treatment, the percentage decrease in AL was highest in plants of the LSIN genotype (−71.4%).

Among individual glucosinolates, significant changes in percentage variation among genotypes were observed in NEO in all treatments except the SA treatment, and these changes in NEO were highest in the LGBS genotype (Table 1 and Table S9; Figure 8C). These percent increases in NEO content in plants of the LGBS genotype were 746.9%, 1122.6%, 685.7%, and 1697.2% for the JA, JAL, SAL, and CL treatments, respectively. In the SA and SAL treatments, differences in percentage variation among genotypes were also significant for SIN, being lowest in plants of the LSIN genotype, which plants showed decreases of −45.7% and −75.4% for the SA and SAL treatments, respectively. In the SAL treatment, the percentage decrease in GIB was higher in plants of the HSIN and LSIN genotypes (−57.4% and −68.8%, respectively) than in plants of the LGBS genotype.

### 2.6. Correlation between Induced Aliphatic and Indolic Glucosinolates

There was a positive correlation between AL and IN content 1 day after JA and SA treatment, 3 days after SA treatment, and 9 days after SAL treatment (Table S10). However, 3 days after treatment, in the CL treatment, there was a negative correlation between AL and IN.

### 2.7. Herbivory and Larval Weight Gain Experiments

**Figure 9:**
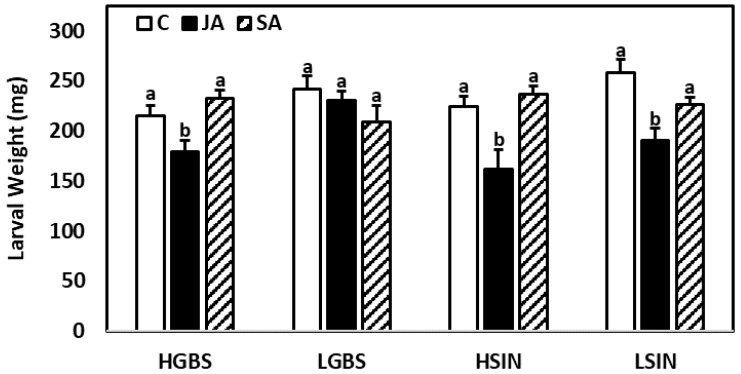
Mean ± SE larval weights after feeding on leaf discs of the different plant genotypes and treatments during 9 days (*n* = 8–10). Post hoc tests with a significance level of *p* ≤ 0.05 were run to compare differences in larval weights within genotypes. Significant differences are shown with different lowercase letters.

For the genotypes HGBS, HSIN, and LSIN, *M. brassicae* larval weights were lower in JA-treated plants than in SA-treated and control plants (Figure 9; Table S11). Larval weights in the SA and control treatments were not significantly different. In plants of the LGBS genotype, differences in larval weights among treatments were not significantly different.

In the HSIN genotype, the percentage of leaf discs with defoliation ≥50% was significantly lower in leaf discs from JA-treated plants than in leaf discs from control plants (Table S12). In the other treatments and genotypes, differences in percentage of leaf discs with defoliation ≥50% were not significantly different. According to this, after JA treatment of plants of the HSIN genotype, herbivory by *M. brassicae* larvae can be significantly reduced.

**Figure 10:**
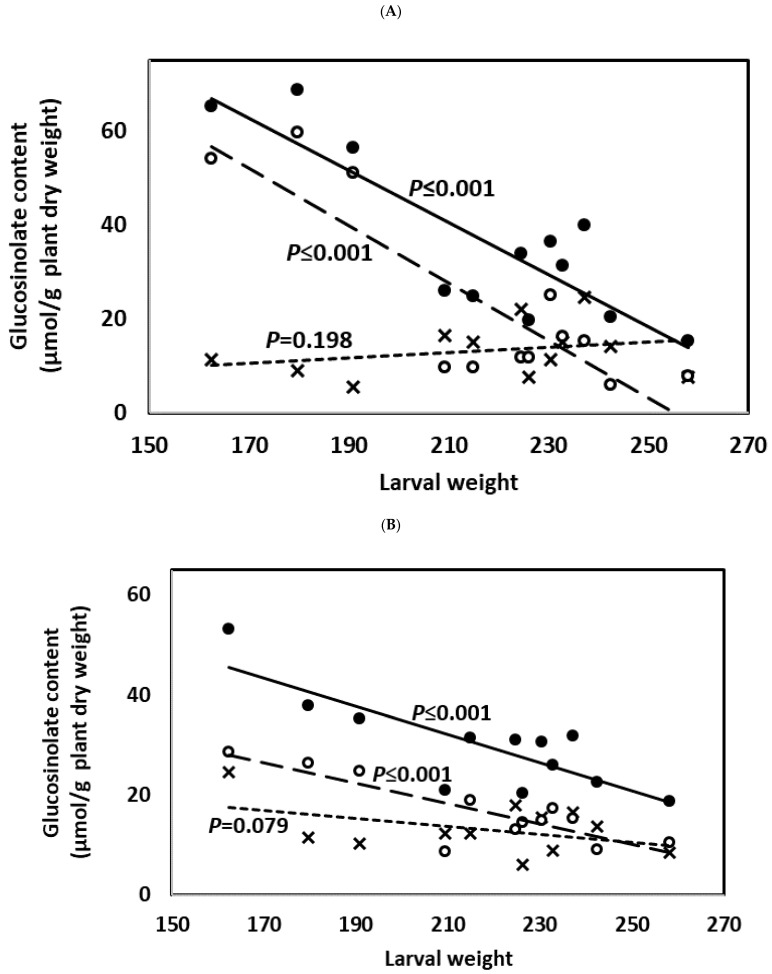
Pearson’s correlations (significance level of *p* ≤ 0.05) between plant glucosinolate content and larval weight at the end of the experiment considering glucosinolate content 3 days (**A**) and 9 days (**B**) after JA and SA treatment. Data used were the glucosinolate averages corresponding to each plant genotype (HGBS, LGBS, HSIN, and LSIN) and treatment (C, JA, and SA) (*n* = 12). Data points are crosses, white circles, and black circles for aliphatic, indolic, and total glucosinolates, respectively. Trends lines are short-dashed, long-dashed, and solid lines for aliphatic, indolic, and total glucosinolates, respectively. Significant differences are shown with different lowercase letters.

**Figure 11:**
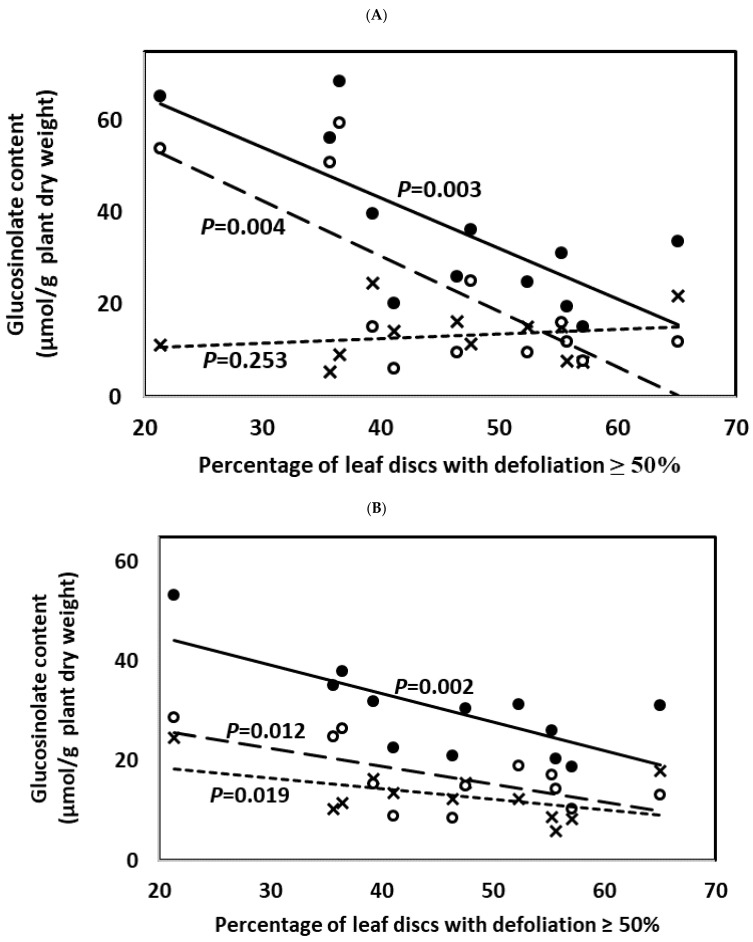
Pearson’s correlations (significance level of *p* ≤ 0.05) between plant glucosinolate content and herbivory (percentage of leaf discs with defoliation ≥50%) considering glucosinolate content 3 days (**A**) and 9 days (**B**) after JA and SA treatment. Data used were the glucosinolate averages corresponding to each plant genotype (HGBS, LGBS, HSIN, and LSIN) and treatment (C, JA, and SA) (*n* = 12). Data points are crosses, white circles, and black circles for aliphatic, indolic, and total glucosinolates, respectively. Trends lines are short-dashed, long-dashed, and solid lines for aliphatic, indolic, and total glucosinolates, respectively. Significant differences are shown with different lowercase letters.

Weight gain in *M. brassicae* larvae and percentage of leaf discs with defoliation ≥50% were negatively correlated with IN content, the type most induced by JA application and by feeding of larvae of *M. brassicae*, and with TO content (Figure 10 and Figure 11; Table S13). This was observed when considering glucosinolate content 3 and 9 days after treatment. Content of AL did not significantly affect weight gain in *M. brassicae* larvae (Table S13A), while the percentage of leaf discs with defoliation ≥50% was only affected by AL content when considering glucosinolate content at the end of the experiment (Table S13B).

## 3. Discussion

Previous studies have shown that there is an induction of glucosinolates as a result of herbivory, JA, and SA application [4,23,27]. Our study is the first to show differences in glucosinolate induction by herbivory and JA and SA application in different genotypes of the same crop through time and combining herbivory with JA and SA treatment. The four genotypes evaluated had been obtained through mass selection from the same variety, and thus they share the same genetic background with differences mostly due to glucosinolate composition [28]. Unlike our study with kale and *M. brassicae*, a previous study showed that low and high glucosinolate genotypes of *B. rapa* did not show changes in glucosinolate profiles as a result of feeding by the specialist root flies *Delia floralis* Fallén and *D. radicum* L. (Diptera: Anthomyiidae), despite differences in the expression of glucosinolate biosynthesis genes [29]. A different study also found differences in glucosinolate induction between wild and domesticated *B. oleracea* as a result of feeding by larvae of the diamondback moth, *Plutella xylostella* L. (Lepidoptera: Plutellidae) [10].

Our research shows that plant genotype affects glucosinolate induction in kale. In our experiments, application of JA and SA, and herbivory by *M. brassicae*, resulted in changes in foliar glucosinolate content that differed among plant genotypes. Application of JA application increased IN and TO contents at the three times tested in plants of the four plant genotypes tested, except in the LGBS genotype, which did not show any significant changes 9 days after JA treatment. The maximum IN and TO contents, which occurred at day 9 after the beginning of the experiment in control plants, were found to occur after 3 days in most of the genotypes in JA-treated and in JAL-treated plants. These findings show that both JA and herbivory induce a rapid accumulation of glucosinolates, particularly NEO and GBS in leaves. Similar results on genotype-specific induction of IN content were found either 2 or 4 days after methyl jasmonate treatment in *B. oleracea* and *B. rapa* [30,31]. Other studies have reported a maximum glucosinolate induction after JA and methyl jasmonate treatment in times varying from 1 to 7 days after treatment [32,33,34].

SA treatment was the most effective to induce content of AL, particularly GIB and SIN. However, induction of AL and TO was only noted for plants of the HGBS and LGBS genotypes 1 day after SA treatment, declining later. These findings agree with those found by a different study that found a significant increase in total glucosinolates in seedlings of *Brassica juncea* (L.) Czern. (Brassicaceae) 1 and 2 days after SA treatment [35]. Aliphatic glucosinolates have been reported to be more stable under different environmental conditions than indolic glucosinolates [36]. These findings were confirmed in our study. The variation in the contents of AL and the individual aliphatic glucosinolates SIN and GIB through time was less marked than the variation in the contents of IN content and the individual indolic glucosinolates GBS and NEO.

Our study also shows that as a result of larval feeding, differences in glucosinolate content among genotypes can change. For example, in the CL, JAL, and SAL treatments, IN contents in plants of the genotype HGBS were no longer higher than in genotype LGBS 3 and 9 days after phytohormone treatment and AL contents were no longer higher in plants of the HSIN genotype than in plants of the LSIN and HGBS genotypes 9 days after treatment.

The percent glucosinolate induction varied with plant genotype, the particular glucosinolate, and time after treatment. The most significant induction in IN content was noticed in the selection made by GBS and it was mostly due to NEO induction. NEO is known to be induced as a result of herbivory, and NEO reached its maximum percent increase 3 days after treatment in the HGBS genotype and 9 days after treatment in the LGBS genotype. IN showed a more significant induction compared to AL, thereby suggesting a prominent role of IN in plant defense response in kale. Similar results were reported previously from other crops like pak choi, *B. rapa* ssp. *chinensis* L., and Chinese cabbage, *B. rapa* ssp. *pekinensis* (Brassicaceae), in which increased accumulation of glucosinolates, particularly indolic ones like NEO, was observed 2–3 days after treatment with methyl jasmonate [34,37]. NEO is known to be induced as a result of herbivory by lepidopteran larvae and plant pathogens [38,39]. Methoxylation modification of indolic glucosinolates is considered very important in plant defense against pathogens and this has been shown with 4-methoxyindol-3-ylmethyl (4-methoxyglucobrassicin, MEOHGBS) [40,41], which was a minor glucosinolate component in the genotypes included in this study. Our study shows that induction of NEO, 1-methoxyindol-3-ylmethyl, another methoxyindol glucosinolate, is very significant as a response to herbivory by *M. brassicae* larvae.

Despite differences in percent induction of glucosinolates among genotypes, there were no significant differences in GBS variation between plants of the HGBS and LGBS genotypes. In the case of SIN, there were only differences in SIN variation between the HSIN and LSIN genotypes in one case (in SA-treated plants 9 days after treatment). This indicates that there was limited trade-off between constitutive and induced GBS and SIN. A partial correlation between constitutive and induced glucosinolates, gene expression, and herbivory has also been found in *Arabidopsis thaliana* (L.) Heynh. (Brassicaceae), suggesting that plant defense goes beyond individual metabolites or genes [42].

Many studies have found that glucosinolate content changes after herbivory in *Brassica* spp. and other plants [4,11,15]. Glucosinolate induction as a result of JA and SA application can also affect plant resistance [43]. Our research shows that plant genotype affects glucosinolate induction in kale after herbivory. In our study, in control plants with *M. brassicae* larvae (CL treatment), IN and TO contents tended to increase compared to control plants, but differences were not significant in all genotypes. The effect of herbivory alone (CL treatment) was not as strong on glucosinolate induction as the effect of JA treatment, indicating that the JA concentration we used was relatively high. We chose this concentration based on a study conducted with *A. thaliana* that used similar concentrations of JA and SA, producing significant induction of glucosinolates and reduction of larval growth in the generalist *Spodoptera exigua* [43]. The combined induction by both JA treatment and herbivory in the JAL treatment caused the largest induction in the glucosinolate NEO 3 days after treatment. However, AL content either was reduced or remained unaltered after herbivory, depending on the genotype. To our knowledge, no other studies have reported on a negative induction of AL content after feeding by generalist larvae. However, we only found a negative correlation between AL and IN content in one case, 3 days after treatment, in the CL treatment, while a positive correlation between AL and IN content was found 1 day after JA and SA treatment, 3 days after SA treatment, and 9 days after treatment in the SAL treatment. Overall, the interaction between previous application of SA and JA and feeding by larvae in the JAL and SAL treatments indicates that there was an enhancement or a cancellation of effects in JA- and SA-treated plants, respectively. Induction of indolic glucosinolates by *M. brassicae* and other generalist larvae has been found in other studies conducted with *A. thaliana* [24,25,44,45,46]. A lack of AL induction by generalist larvae has also been found in other studies conducted with *A. thaliana* [24,25,45,46,47], while a positive induction of AL in *tgg1 tgg2* mutant plants of *A. thaliana* that lack the major myrosinases was also reported by Badenes-Pérez et al. [24]. By contrast, feeding by larvae of the specialist *P. xylostella* resulted in negative induction of aliphatic and indolic glucosinolates in certain genotypes of *B. oleracea* and *A. thaliana*, respectively [10,24].

Our glucosinolate results refer particularly to the top two leaves of plants that were 6 weeks old at the beginning of the experiment, and through 9 days later. Over the 9-day length of the experiment, content of IN, TO, GBS, and NEO increased in control plants of the genotypes HGBS, LGBS, and HSIN, while in control plants of the genotype LSIN only SIN changed (increased). Plant age and leaf age within a plant are known to greatly affect glucosinolate content [48,49]. As glucosinolates can be induced as a result of herbivory, glucosinolate content is likely to have changed during the larval feeding and weight gain experiments compared to the glucosinolate data presented here for intact plants.

*Mamestra brassicae* and other generalist noctuids can be important pests in crops of the family Brassicaceae [50,51,52,53]. Larvae of *M. brassicae* have been found to cope well with some plant defense compounds, such as the triterpenoid saponins present in some *Barbarea* spp. (Brassicaceae) that are resistant to other herbivores [54,55,56,57]. In the case of glucosinolates, aliphatic, indolic, and benzenic glucosinolates have been shown to have detrimental effects on *M. brassicae* larvae [9,11,12,13]. In this study, we found a negative correlation between larval weight gain in *M. brassicae* and IN and TO content, but not with AL content. This indicates that plant varieties with higher content of indolic glucosinolates, such as glucobrassicin, may be better equipped to defend themselves against herbivory by generalist herbivores like *M. brassicae*. Other studies with *M. brassicae* and other generalist herbivores, like *Spodoptera exigua* Hübner (Lepidoptera: Noctuidae), have found a negative correlation between aliphatic glucosinolate content and larval weight gain [9,11,58]. Besides glucosinolates, myrosinase activity can also cause resistance to *M. brassicae* [12,26]. However, using plant varieties with high glucosinolate content may increase susceptibility to specialist herbivores like *P. xylostella* [8]. For example, females of *P. xylostella* preferred ovipositing on *B. vulgaris* plants with a 20% higher content of the dominant glucosinolate glucobarbarin [59]. In this study, in the case of some treatments, like the JA and JAL treatments 3 days after treatment, we detected percentage increases in indolic glucosinolates that were much larger than 20%. Besides lepidopteran larvae, plant pathogens can also change glucosinolate content in Brassicaceae and changes can be variable depending on the plant species and cultivar [60,61,62,63]. For example, IN increased in plants of two *B. rapa* cultivars after infection with *Leptosphaeria maculans* and *Fusarium oxysporum*, but TO and AL increased in one cultivar and decreased in the other [60].

Application of methyl jasmonate also reduced herbivory by *M. brassicae* in ragwort, *Jacobaea* spp. (Asteraceae), and an increase in jacaranone, asparagine, threonine, isoleucine, and citric acid was associated to reduced herbivory after methyl jasmonate application [64]. Jacaranone has not been found in Brassicaceae, but some of these amino acids and citric acid are quite ubiquitously present in plants, so they could also be associated with reduced herbivory by *M. brassicae* in *B. oleracea* plants. Further research is necessary to study if other compounds besides glucosinolates might have played a role in the reduction of *M. brassicae* larval weights and herbivory that we detected in this study. In our study, reduced larval weights of *M. brassicae* were observed in JA-treated plants of the HGBS, HSIN, and LSIN genotypes, but a significant reduction of herbivory by *M. brassicae* larvae was only observed in JA-treated plants of the HSIN genotype. This indicates that, among the four genotypes that we tested, the HSIN genotype may be the best to conduct additional studies on the induction of plant defenses in kale.

## 4. Materials and Methods

### 4.1. Plants and Insects Used in the Experiments

Plants of kale, *B. oleracea* var. *acephala*, originated from a divergent selection program started in 2006 using kale population MBG-BRS0062 from the Brassica seedbank at Misión Biológica de Galicia (CSIC), northwestern Spain. The four kale genotypes tested had high (HSIN) and low sinigrin (LSIN) content and high (HGBS) and low glucobrassicin (LGBS) content [27]. Plants were grown in 18.7 cm diameter pots. Plants were 6 weeks old at the beginning of the experiments. *Mamestra brassicae* eggs were provided by the Centre de Recherches de Versailles (Versailles, France). After egg hatching, larvae were fed fresh cabbage leaves and were reared in plastic boxes in the laboratory (21 ± 3 °C, 65 ± 5 RH, and natural photoperiod).

### 4.2. Application of Phytohormones on Plants

A hand pump sprayer was used to apply approximately 14.3 mL/plant of either JA or SA in 0.5 mM concentrations including 0.1% Tween 20 (Sigma-Aldrich Chemie GmbH, Schnelldorf, Germany). Control plants were sprayed only with 0.1% Tween 20 solution. Treatment application on each individual plant lasted until runoff of the solution on plant foliage.

### 4.3. Interaction between Phytohormone and Herbivore Induction

One day after the application of phytohormones, 5 larvae per plant were placed on the leaves of 10 plants of each genotype and treatment. Thus, besides the control (C), JA, and SA treatments, three more treatments were included: control with larvae (CL), JA with larvae (JAL), and SA with larvae (SAL).

### 4.4. Analysis of Glucosinolates in Plants

To determine glucosinolate content, we harvested the top two leaves of each plant 1, 3, and 9 days after the application of phytohormones (3 and 9 days after the application of phytohormones in the case of the treatments with larvae, CL, JAL, and SAL). After freezing and freeze-drying these samples, glucosinolate content was analyzed as in Sotelo et al. [27]. Glucosinolate analysis was first used to determine the glucosinolate content in the control plants of each genotype. Thereafter, we focused on total aliphatic (AL), total indolic (IN), and total glucosinolate content (TO).

### 4.5. Herbivory and Larval Weight Gain Experiments

For each plant genotype and treatment, 10 third-instar larvae were individually placed in petri dishes of 9 cm diameter. Larvae were fed with leaf discs of 35 mm diameter collected from plants of the different treatments over a period of 9 days. Two middle leaves were used, i.e., any leaves except the top two ones and the bottom one. Larvae were inspected daily, and leaf discs were replaced by fresh ones on days 2 through 8. Feeding by larvae was visually assessed as having defoliated either ≥50% or <50% of the leaf disc. The total number of leaf discs with defoliation ≥50% were summed up at the end of the experiment, comparing the total to a potential maximum of one leaf disc for each of the 7 days in which herbivory was assessed. A percentage of leaf discs with defoliation ≥50% was calculated as the number of leaf discs with defoliation ≥50% divided by the potential maximum of one leaf disc per day multiplied by the number of replicates, which was 10 if none of the larvae had died from the beginning to the end of the experiment. The weights of larvae were measured at the beginning and end of the experiment (day 9) and the weight at day 9 was used to compare the effect of the different treatments and plant genotypes on larval weight gain.

### 4.6. Statistical Analyses

Differences in glucosinolate content among plants of each genotype, phytohormone and herbivory treatment, and time (days after phytohormone treatment) were analyzed using either one-way ANOVA, if data were parametric, followed by either LSD or Tamhane, or Kruskal–Wallis and Mann–Whitney tests (*p* ≤ 0.05) using SPSS^®^ version 26 (IBM Corp., Armonk, NY, USA). In the post hoc analysis after the Kruskal–Wallis tests, the *p*-value that we used was the one with the adjusted significance, adjusted by the Bonferroni correction for multiple tests. Percentages of leaf discs consumed over a period of 9 days, were analyzed using a one-tailed, two-sample test of proportions using STATA^®^ version 15.1 with significance at *p* ≤ 0.05. Differences in larval weights among the different treatments and species were analyzed using Kruskal–Wallis tests, using the significance adjusted by the Bonferroni correction in post hoc analysis. Correlations between aliphatic and indolic glucosinolate induction were performed using one-tailed Spearman’s rho correlations with SPSS^®^. Correlations between leaf disc consumption and larval weights with glucosinolate content were performed using one-tailed Pearson correlations with SPSS^®^.

## Figures and Tables

**Table 1:** Mean ± SE percentage change in glucosinolate content (%) for each genotype and treatment one day after the application of the phytohormones (*n* = 3–10). The treatments are jasmonic acid (JA), salicylic acid (SA), JA with *Mamestra brassicae* larvae (JAL), SA with *M. brassicae* larvae (SAL), and control with *M. brassicae* larvae (CL). The genotypes are high in glucobrassicin (HGBS), low in glucobrassicin (LGBS), high in sinigrin (HSIN), and low in sinigrin (LSIN). The glucosinolates shown are glucoiberin (GIB), sinigrin (SIN), glucobrassicin (GBS), neoglucobrassicin (NEO), total aliphatic (AL), total indolic (IN), and total glucosinolates (TO). For each time (days after treatment) and treatment, means within a column followed by different letters show significant differences (*p* ≤ 0.05) among genotypes. Replication was *n* = 7–10, *n* = 5–10, and *n* = 3–5 for 1, 3, and 9 days after treatment, respectively.

Days after Treat.	Tr.	Genotype	GIB	SIN	GBS	NEO	AL	IN	TO
1	JA	HGBS	69.3 ± 30.7 a	43.4 ± 17.5 a	195.7 ± 41.1 a	2198.0 ± 492.8 a	51.1 ± 17.7 a	292.3 ± 46.4 a	142.0 ± 21.7 a
		LGBS	60.8 ± 26.2 a	48.5 ± 29.8 a	320.9 ± 70.0 a	655.2 ± 139.4 b	50.8 ± 27.4 a	335.7 ± 64.5 a	132.7 ± 29.5 a
		HSIN	24.2 ± 18.6 a	−29.1 ± 10.1 a	171.8 ± 30.7 a	1584.8 ± 260.9 a	−17.2 ± 10.7 b	216.6 ± 29.5 ab	52.1 ± 13.3 b
		LSIN	−46.4 ± 6.9 b	37.4 ± 58.4 a	147.6 ± 36.6 a	230.6 ± 87.7 b	−12.6 ± 24.8 b	151.9 ± 32.1 b	65.0 ± 24.1 b
	SA	HGBS	97.2 ± 25.1 a	38.5 ± 17.8 a	29.3 ± 21.1 a	94.5 ± 46.1 ab	58.9 ± 11.3 a	37.7 ± 19.0 ab	51.3 ± 8.6 a
		LGBS	82.6 ± 21.7 a	77.1 ± 33.7 a	47.5 ± 20.3 a	171.8 ± 51.5 b	78.2 ± 23.6 a	51.6 ± 19.5 a	70.9 ± 19.7 a
		HSIN	10.0 ± 12.2 b	13.3 ± 16.7 a	−6.5 ± 11.8 a	5.7 ± 25.5 a	11.0 ± 12.1 b	−5.0 ± 34.4 b	6.2 ± 10.6 b
		LSIN	−18.4 ± 13.5 b	38.5 ± 28.3 a	5.5 ± 12.2 a	−9.7 ± 34.7 a	−0.7 ± 16.8 b	−0.3 ± 10.6 b	−0.5 ± 12.1 b
3	JA	HGBS	−32.3 ± 11.7 a	−43.7 ± 10.1 a	355.3 ± 40.1 a	2837.5 ± 777.7 a	−40.4 ± 9.8 a	514.4 ± 54.0 ac	176.2 ± 21.9 a
		LGBS	−26.6 ± 14.2 a	−19.1 ± 15.3 a	300.4 ± 50.5 a	498.6 ± 222.2 b	−20.1 ± 13.1 a	313.6 ± 49.4 b	79.7 ± 19.1 b
		HSIN	−42.8 ± 8.9 a	−49.7 ± 8.1 a	274.2 ± 55.4 a	1363.6 ± 292.9 ab	−48.6 ± 7.0 a	357.1 ± 46.6 ab	93.6 ± 18.2 b
		LSIN	−13.6 ± 14.6 a	−49.1 ± 13.6 a	376.1 ± 71.3 a	2145.0 ± 625.1 a	−27.9 ± 10.5 a	558.1 ± 75.1 c	269.9 ± 37.0 c
	JAL	HGBS	−3.1 ± 15.7 a	−54.2 ± 14.5 a	568.9 ± 86.5 a	3655.5 ± 709.6 a	−39.2 ± 14.6 a	763.2 ± 113.1 a	273.8 ± 50.0 a
		LGBS	−4.0 ± 18.3 a	−27.1 ± 18.7 a	545.6 ± 132.7 a	1039.6 ± 257.5 a	−18.0 ± 8.8 a	570.8 ± 124.4 a	158.1 ± 33.2 b
		HSIN	−37.3 ± 19.1 a	−37.1 ± 13.4 a	118.2 ± 62.5 b	2051.9 ± 669.5 a	−38.8 ± 9.0 a	265.3 ± 27.9 b	67.7 ± 8.4 c
		LSIN	−12.7 ± 24.0 a	−44.4 ± 13.1 a	201.3 ± 74.2 b	3106.3 ± 933.5 a	−13.7 ± 16.7 a	492.8 ± 71.3 ab	243.8 ± 32.1 ab
	SA	HGBS	−10.7 ± 12.4 a	16.8 ± 15.3 a	71.4 ± 26.5 a	64.0 ± 45.9 a	26.1 ± 27.4 a	83.9 ± 27.7 a	82.3 ± 56.1 a
		LGBS	10.9 ± 17.2 a	16.3 ± 14.7 a	55.2 ± 17.0 a	10.4 ± 19.6 a	15.2 ± 11.4 a	60.1 ± 15.8 a	28.6 ± 10.0 a
		HSIN	24.0 ± 14.3 a	10.5 ± 10.3 a	27.1 ± 10.3 a	−3.8 ± 30.4 a	12.5 ± 7.0 a	29.0 ± 11.3 a	18.2 ± 7.2 a
		LSIN	−8.9 ± 20.7 a	8.5 ± 15.8 a	50.4 ± 35.1 a	49.1 ± 36.7 a	2.1 ± 15.2 a	52.9 ± 35.9 a	28.7 ± 23.3 a
	SAL	HGBS	−3.9 ± 17.5 a	7.0 ± 12.2 a	129.1 ± 38.0 a	458.4 ± 75.9 a	3.8 ± 11.1 a	150.7 ± 36.1 a	60.9 ± 16.9 a
		LGBS	34.2 ± 10.4 a	33.2 ± 26.2 a	276.3 ± 76.6 a	662.8 ± 168.0 a	33.6 ± 17.0 a	300.2 ± 72.8 a	113.3 ± 31.5 a
		HSIN	22.0 ± 14.9 a	−16.2 ± 20.1 a	90.3 ± 26.0 a	1397.6 ± 642.7 a	−8.6 ± 16.2 a	190.9 ± 64.9 a	61.3 ± 14.9 a
		LSIN	2.7 ± 15.1 a	−14.9 ± 32.8 a	173.1 ± 84.3 a	1314.8 ± 718.6 a	−5.4 ± 17.3 a	286.9 ± 50.6 a	143.8 ± 26.1 a
	CL	HGBS	−9.7 ± 7.5 a	−53.8 ± 6.2 a	−11.6 ± 15.3 a	558.6 ± 164.9 a	−40.9 ± 3.8 a	25.9 ± 22.3 a	−12.9 ± 9.0 a
		LGBS	24.6 ± 6.6 a	−16.2 ± 10.2 a	130.0 ± 27.0 b	467.1 ± 73.6 a	−18.3 ± 7.0 a	151.3 ± 27.6 b	32.4 ± 7.9 b
		HSIN	−47.8 ± 14.5 a	−42.2 ± 17.9 a	113.4 ± 23.4 b	439.3 ± 224.6 a	−44.4 ± 16.0 a	137.3 ± 34.7 ab	20.6 ± 12.3 ab
		LSIN	−23.8 ± 13.5 a	−35.7 ± 10.9 a	268.7 ± 69.1 c	740.9 ± 216.6 a	−28.9 ± 6.6 a	314.6 ± 71.6 c	146.1 ± 39.2 c
9	JA	HGBS	55.1 ± 34.2 a	−28.9 ± 21.1 a	21.5 ± 13.8 a	177.1 ± 51.3 a	−7.0 ± 14.4 a	39.6 ± 12.2 a	21.2 ± 11.9 a
		LGBS	63.9 ± 45.7 a	−6.7 ± 19.6 a	42.6 ± 30.9 a	746.9 ± 256.8 b	14.2 ± 20.0 a	65.7 ± 25.5 a	34.7 ± 18.8 ab
		HSIN	25.1 ± 14.9 a	45.4 ± 16.7 a	94.8 ± 23.0 a	252.1 ± 103.1 a	37.1 ± 11.1 a	118.5 ± 20.9 a	71.4 ± 15.0 bc
		LSIN	28.0 ± 35.2 a	10.8 ± 32.9 a	142.8 ± 44.8 a	116.4 ± 38.4 a	22.2 ± 29.7 a	138.8 ± 41.3 a	86.7 ± 19.7 c
	JAL	HGBS	−42.8 ± 30.5 a	−61.7 ± 9.7 a	122.3 ± 15.3 a	226.7 ± 36.0 a	−57.1 ± 14.1 a	132.9 ± 12.2 a	57.7 ± 12.5 a
		LGBS	−61.0 ± 11.0 a	−69.4 ± 10.4 a	211.9 ± 42.7 a	1122.6 ± 394.2 b	−66.9 ± 10.3 a	236.7 ± 39.0 a	54.0 ± 13.4 a
		HSIN	−36.8 ± 21.7 a	−73.5 ± 10.6 a	130.2 ± 47.4 a	179.1 ± 54.1 a	−63.9 ± 7.9 a	132.7 ± 40.0 a	21.4 ± 38.0 a
		LSIN	−79.1 ± 4.0 a	−44.3 ± 38.7 a	207.8 ± 68.4 a	316.0 ± 130.4 a	−62.3 ± 20.0 a	221.2 ± 76.8 a	94.5 ± 51.2 a
	SA	HGBS	−17.6 ± 17.0 a	−32.6 ± 6.5 ac	−17.8 ± 15.6 a	40.7 ± 33.2 a	−29.0 ± 7.5 a	−8.9 ± 9.7 a	−16.9 ± 6.3 a
		LGBS	−6.2 ± 19.6 a	−14.7 ± 5.3 ab	−12.7 ± 30.1 a	128.8 ± 48.3 a	−9.1 ± 3.8 a	−4.3 ± 28.0 a	−7.2 ± 13.1 a
		HSIN	−31.2 ± 12.8 a	1.7 ± 10.0 b	21.2 ± 17.9 a	−8.1 ± 23.5 a	−8.3 ± 10.0 a	17.2 ± 17.0 a	2.4 ± 9.7 a
		LSIN	−13.2 ± 16.9 a	−45.7 ± 12.6 c	9.4 ± 17.7 a	189.6 ± 110.3 a	−28.9 ± 6.0 a	38.4 ± 13.8 a	8.3 ± 9.0 a
	SAL	HGBS	−34.6 ± 14.5 ab	−64.5 ± 11.7 a	13.6 ± 11.5 a	68.6 ± 42.5 a	−59.2 ± 10.4 ac	19.4 ± 10.9 a	−10.9 ± 10.2 a
		LGBS	−12.5 ± 11.8 a	−35.7 ± 11.2 bc	121.8 ± 44.2 a	685.7 ± 162.8 b	−28.5 ± 9.1 b	138.2 ± 47.0 b	37.9 ± 23.5 a
		HSIN	−57.4 ± 13.9 b	−40.6 ± 4.9 ab	124.7 ± 24.3 a	124.3 ± 62.2 a	−42.9 ± 6.3 ab	123.9 ± 21.7 b	27.4 ± 11.8 a
		LSIN	−68.8 ± 7.9 b	−75.4 ± 6.0 c	91.1 ± 28.6 a	193.3 ± 93.5 a	−71.4 ± 3.0 c	10.6 ± 27.3 ab	26.8 ± 16.0 a
	CL	HGBS	−70.1 ± 7.3 a	−80.7 ± 1.0 a	24.3 ± 14.3 a	71.5 ± 46.4 a	−63.6 ± 2.2 a	30.2 ± 14.4 a	−6.9 ± 7.9 a
		LGBS	−40.4 ± 11.1 a	−83.3 ± 3.3 a	117.6 ± 42.6 a	1697.2 ± 577.7 b	−59.8 ± 5.7 a	170.0 ± 50.0 a	31.7 ± 20.9 a
		HSIN	−59.2 ± 8.0 a	−78.2 ± 3.2 a	97.5 ± 33.0 a	437.9 ± 103.4 a	−52.3 ± 7.0 a	138.7 ± 37.2 a	28.2 ± 14.5 a
		LSIN	−1.0 ± 20.4 b	−67.8 ± 17.1 a	2.6 ± 53.8 a	370.9 ± 206.5 a	−7.8 ± 29.0 b	63.3 ± 48.1 a	31.5 ± 35.7 a

## Data Availability

The data that support the findings of this study are available from the corresponding author upon reasonable request.

## Supplementary Materials

The following are available online at https://www.mdpi.com/article/10.3390/plants10091951/s1, Table S1: Glucosinolate content for each time (days after treatment), genotype, and treatment (*n* = 7–10). The treatments are control (C), jasmonic acid (JA), salicylic acid (SA), control with *M. brassicae* larvae (CL), JA with M. brassicae larvae (JAL), and SA with *M. brassicae* larvae (SAL). The genotypes are high in glucobrassicin (HGBS), low in glucobrassicin (LGBS), high in sinigrin (HSIN), and low in sinigrin (LSIN). The glucosinolates shown are progoitrin (PRO), glucoiberverin (GIV), 4-hydroxyglucobrassicin (OHGBS), 4-methoxyglucobrassicin (MEOHGBS), neoglucobrassicin (NEO), and gluconasturtiin (GNT). Replication was *n* = 7–10, *n* = 5–10, and *n* = 3–5 for 1, 3, and 9 days after treatment, respectively; Table S2: Differences in glucosinolate content across times (days after treatment with JA and SA). Test statistic and *p*-values of ANOVA or Kruskall–Wallis test shown to compare differences in glucosinolate content among times (1, 3, and 9 days after treatment in the case of the treatments C, JA, and SA, and 3 and 9 days after treatment in the case of CL, JAL, and SAL) within the same genotype. Significant *p*-values (*p* ≤ 0.05) are shown in bold type; Table S3: Mean ± SE glucosinolate content (µmol g^−1^ plant dry weight) for each treatment and genotype after the application of phytohormones, 1, 3, and 9 days after treatment. The treatments are control (C), jasmonic acid (JA), salicylic acid (SA), control with M. brassicae larvae (CL), JA with M. brassicae larvae (JAL), and SA with M. brassicae larvae (SAL). The genotypes are high in glucobrassicin (HGBS), low in glucobrassicin (LGBS), high in sinigrin (HSIN), and low in sinigrin (LSIN). The glucosinolates shown are glucoiberin (GIB), sinigrin (SIN), glucobrassicin (GBS), neoglucobrassicin (NEO), total aliphatic (AL), total indolic (IN), and total glucosinolates (TO). The less abundant glucosinolates progoitrin (PRO), glucoiberverin (GIV), 4-hydroxyglucobrassicin (OHGBS), 4-methoxyglucobrassicin (MEOHGBS), and gluconasturtiin (GNT) are not shown here, but are shown as supplementary data. For each genotype and treatment, means within a column followed by different letters show significant differences (*p* ≤ 0.05) in time (days after treatment). Replication was *n* = 7–10, *n* = 5–10, and *n* = 3–5 for 1, 3, and 9 days after treatment, respectively; Table S4: Effect of JA, SA, CL, JAL, and SAL treatments on glucosinolate content in the different genotypes, 1, 3, and 9 days after treatment with JA and SA. Test statistic and *p*-values of ANOVA or Kruskall–Wallis test shown to compare differences in glucosinolate content among treatments within the same genotype. Treatments included in the comparisons among treatments are C, JA, and SA (also CL for 3 and 9 days after treatment) (A), and JA compared to JAL and SA compared to SAL (3 and 9 days after treatment) (B). Significant *p*-values (*p* ≤ 0.05) are shown in bold type; Table S5: Mean ± SE glucosinolate content (µmol g^−1^ plant dry weight) for each treatment and genotype after the application of phytohormones. The treatments are control (C), jasmonic acid (JA), salicylic acid (SA), and control with *M. brassicae* larvae (CL). The genotypes are high in glucobrassicin (HGBS), low in glucobrassicin (LGBS), high in sinigrin (HSIN), and low in sinigrin (LSIN). The glucosinolates shown are glucoiberin (GIB), sinigrin (SIN), glucobrassicin (GBS), neoglucobrassicin (NEO), total aliphatic (AL), total indolic (IN), and total glucosinolates (TO). The less abundant glucosinolates progoitrin (PRO), glucoiberverin (GIV), 4-hydroxyglucobrassicin (OHGBS), 4-methoxyglucobrassicin (MEOHGBS), and gluconasturtiin (GNT) are not shown here, but are shown as supplementary data. For each time (days after treatment) and genotype, means within a column followed by different letters show significant treatment differences (*p* ≤ 0.05) among genotypes. Replication was *n* = 7–10, *n* = 5–10, and *n* = 3–5 for 1, 3, and 9 days after treatment, respectively; Table S6: Mean ± SE glucosinolate content (µmol g^−1^ plant dry weight) in kale genotypes after the application of phytohormones to genotypes high in glucobrassicin (HGBS), low in glucobrassicin (LGBS), high in sinigrin (HSIN), and low in sinigrin (LSIN) (*n* = 3–10). The treatments are jasmonic acid (JA), salicylic acid (SA), JA with M. brassicae larvae (JAL), and SA with M. brassicae larvae (SAL). The glucosinolates shown glucoiberin (GIB), sinigrin (SIN), glucobrassicin (GBS), neoglucobrassicin (NEO), total aliphatic (AL), total indolic (IN), and total glucosinolates (TO). The less abundant glucosinolates progoitrin (PRO), glucoiberverin (GIV), 4-hydroxyglucobrassicin (OHGBS), 4-methoxyglucobrassicin (MEOHGBS), and gluconasturtiin (GNT) are shown as supplementary data. For each time (days after treatment) and genotype, means within a column followed by different letters show significant treatment differences (*p* ≤ 0.05) among genotypes. Replication was *n* = 7–10, *n* = 5–10, and *n* = 3–5 for 1, 3, and 9 days after treatment, respectively; Table S7: Changes in glucosinolate content among genotypes under C, JA, SA, CL, JAL treatments 1, 3, and 9 days after treatment with JA and SA. Test statistic and *p*-values of ANOVA or Kruskall–Wallis test shown to compare differences in glucosinolate content among genotypes subject to the same treatment. Significant *p*-values (*p* ≤ 0.05) are shown in bold type; Table S8: Mean ± SE glucosinolate content (µmol g^−1^ plant dry weight) for each treatment and genotype after the application of phytohormones (*n* = 3–10). The treatments are control (C), jasmonic acid (JA), salicylic acid (SA), control with M. brassicae larvae (CL), JA with M. brassicae larvae (JAL), and SA with M. brassicae larvae (SAL). The genotypes are high in glucobrassicin (HGBS), low in glucobrassicin (LGBS), high in sinigrin (HSIN), and low in sinigrin (LSIN). The glucosinolates shown are glucoiberin (GIB), sinigrin (SIN), glucobrassicin (GBS), neoglucobrassicin (NEO), total aliphatic (AL), total indolic (IN), and total glucosinolates (TO). The less abundant glucosinolates progoitrin (PRO), glucoiberverin (GIV), 4-hydroxyglucobrassicin (OHGBS), 4-methoxyglucobrassicin (MEOHGBS), and gluconasturtiin (GNT) are not shown here, but are shown as supplementary data. For each time (days after treatment) and treatment, means within a column followed by different letters show significant differences (*p* ≤ 0.05) among genotypes. Replication was *n* = 7–10, *n* = 5–10, and *n* = 3–5 for 1, 3, and 9 days after treatment, respectively; Table S9: Effect of JA, SA, CL, JAL, and SAL treatments on glucosinolate content in the different genotypes, 1, 3, and 9 days after treatment with JA and SA. Test statistic and *p*-values of ANOVA or Kruskall–Wallis test shown to compare differences in glucosinolate content (percentages, compared to the control within each genotype) among genotypes subject to the same treatment. Significant *p*-values (*p* ≤ 0.05) are shown in bold type; Table S10: Significance of correlations between aliphatic (AL) and indolic (IN) glucosinolate in induced plants. Data used in the correlations included all glucosinolate data from the four plant genotypes (HGBS, LGBS, HSIN, and LSIN) for each of the treatments, 1, 3 and 9 days after JA and SA treatment (*n* = 39–40) and 3 and 9 days after CL, JAL, and SAL treatments began (*n* = 19–20). Significant *p*-values (*p* ≤ 0.05) of one-tailed Spearman’s rho correlation are shown in bold type; Table S11: Differences in larval weights after feeding on leaf discs of the different plant genotypes and treatments during 9 days (*n* = 8–10). *p*-values from Mann-Whitney U tests. Significant *p*-values (*p* ≤ 0.05) are shown in bold type; Table S12: Comparison of the percentage of leaf discs with defoliation ≥50% as a result of larval feeding under control (C), jasmonic acid (JA), and salicylic acid (SA) treatments. Significant *p*-values (*p* ≤ 0.05) of one-tailed two-sample tests of proportions are shown in bold type; and Table S13. Significance of correlations between plant glucosinolate content and larval weight at the end of the experiment (A) and between plant glucosinolate content and percentage of leaf discs with defoliation ≥ 50% (B). Correlations are shown for glucosinolate content 3 days and 9 days after JA and SA treatment. Data used were the glucosinolate averages corresponding to each plant genotype (HGBS, LGBS, HSIN, and LSIN) and treatment (C, JA, and SA) (*n* = 12). Three different classes of glucosinolates were distinguished, aliphatic (AL), indolic (IN), and total (TO). Significant *p*-values (*p* ≤ 0.05) are shown in bold type.
