# Supplementary material for: Glucosinolate Induction and Resistance to the Cabbage Moth, Mamestra brassicae, Differs among Kale Genotypes with High and Low Content of Sinigrin and Glucobrassicin
Source: Plants (Basel). 2021 Sep 18;10(9):1951. doi: 10.3390/plants10091951 (PMC8469716; doi:10.3390/plants10091951)
Supplement: Supplementary file 1 [file plants-10-01951-s001.zip › plants-1363399-supplementary.pdf]

Glucosinolate induction and resistance to the cabbage moth, *Mamestra brassicae*, differs among kale genotypes with high and low content of sinigrin and glucobrassicin

Francisco Rubén Badenes-Pérez[1] and María Elena Cartea[2]

[1] *Instituto de Ciencias Agrarias, Consejo Superior de Investigaciones Científicas, 28006 Madrid, Spain*

[2] *Misión Biológica de Galicia, Consejo Superior de Investigaciones Científicas, 36080 Pontevedra, Spain*

Author for correspondence:

Francisco Rubén Badenes-Pérez

Tel: +34 917452500

Email: fr.badenes@ csic.es

Table S1. Glucosinolate content for each time (days after treatment), genotype, and treatment (n=7-10). The treatments are control (C), jasmonic acid (JA), salicylic acid (SA), control with *M. brassicae* larvae (CL), JA with *M. brassicae* larvae (JAL), and SA with *M. brassicae* larvae (SAL). The genotypes are high in glucobrassicin (HGBS), low in glucobrassicin (LGBS), high in sinigrin (HSIN), and low in sinigrin (LSIN). The glucosinolates shown are progoitrin (PRO), glucoiberverin (GIV), 4-hydroxyglucobrassicin (OHGBS), 4-methoxyglucobrassicin (MEOHGBS), neoglucobrassicin (NEO), and gluconasturtiin (GNT). Replication was n=7-10, n=5-10, and n=3-5 for 1, 3, and 9 days after treatment, respectively. 28

Days after treatment	Genotype	Treatment	PRO	GIV	OHGBS	MEOHGBS	GNT
1	**HGBS**	**C**	1.1±0.1	0.0±0.0	0.3±0.1	0.1±0.0	0.0±0.0
1	**LGBS**	**C**	0.3±0.1	0.0±0.0	0.1±0.1	0.0±0.0	0.0±0.0
1	**HSIN**	**C**	0.5±0.2	0.0±0.0	0.3±0.1	0.2±0.0	0.0±0.0
1	**LSIN**	**C**	0.1±0.1	0.0±0.0	0.3±0.1	0.1±0.0	0.0±0.0
1	**HGBS**	**JA**	0.2±0.1	0.0±0.0	1.4±0.2	0.0±0.0	0.8±0.1
1	**LGBS**	**JA**	0.2±0.1	0.0±0.0	0.9±0.2	0.0±0.0	0.5±0.2
1	**HSIN**	**JA**	0.2±0.2	0.0±0.0	0.4±0.1	0.2±0.0	0.0±0.0
1	**LSIN**	**JA**	0.6±0.5	0.0±0.0	0.3±0.1	0.2±0.1	0.1±0.1
1	**HGBS**	**SA**	0.6±0.3	0.0±0.0	0.4±0.1	0.4±0.1	0.0±0.0
1	**LGBS**	**SA**	0.4±0.1	0.0±0.0	0.1±0.0	0.0±0.0	0.0±0.0
1	**HSIN**	**SA**	0.3±0.1	0.0±0.0	0.3±0.1	0.2±0.0	0.0±0.0
1	**LSIN**	**SA**	0.0±0.0	0.0±0.0	0.1±0.1	0.1±0.0	0.0±0.0
3	**HGBS**	**C**	0.0±0.0	0.0±0.0	0.0±0.0	0.1±0.0	0.0±0.0
3	**LGBS**	**C**	0.0±0.0	0.0±0.0	0.0±0.0	0.1±0.0	0.0±0.0
3	**HSIN**	**C**	0.6±0.3	0.0±0.0	0.0±0.0	0.2±0.0	0.0±0.0
3	**LSIN**	**C**	0.0±0.0	0.0±0.0	0.0±0.0	0.1±0.0	0.0±0.0
3	**HGBS**	**CL**	0.0±0.0	0.0±0.0	0.0±0.0	0.2±0.1	0.5±0.1
3	**LGBS**	**CL**	0.2±0.2	0.0±0.0	0.0±0.0	0.2±0.0	0.0±0.0
3	**HSIN**	**CL**	0.2±0.2	0.4±0.1	0.1±0.1	0.2±0.0	0.0±0.0
3	**LSIN**	**CL**	0.0±0.0	0.0±0.0	0.0±0.0	0.1±0.0	0.1±0.1
3	**HGBS**	**JA**	0.0±0.0	0.0±0.0	0.4±0.2	0.3±0.0	0.0±0.0

3	**LGBS**	**JA**	0.3±0.2	0.0±0.0	0.2±0.1	0.3±0.0	0.0±0.0
3	**HSIN**	**JA**	0.1±0.1	0.0±0.0	0.4±0.2	0.3±0.0	0.0±0.0
3	**LSIN**	**JA**	0.2±0.1	0.0±0.0	0.6±0.3	0.2±0.0	0.0±0.0
3	**HGBS**	**JAL**	0.0±0.0	0.0±0.0	0.1±0.1	0.4±0.1	0.0±0.0
3	**LGBS**	**JAL**	0.0±0.0	0.0±0.0	0.0±0.0	0.3±0.0	0.0±0.0
3	**HSIN**	**JAL**	0.0±0.0	0.0±0.0	0.0±0.0	0.3±0.0	0.0±0.0
3	**LSIN**	**JAL**	0.0±0.0	0.0±0.0	0.1±0.1	0.4±0.1	0.0±0.0
3	**HGBS**	**SA**	0.1±0.1	0.0±0.0	0.5±0.2	0.3±0.0	0.0±0.0
3	**LGBS**	**SA**	0.1±0.1	0.0±0.0	0.5±0.1	0.2±0.0	0.0±0.0
3	**HSIN**	**SA**	0.3±0.1	0.0±0.0	0.5±0.1	0.2±0.0	0.0±0.0
3	**LSIN**	**SA**	0.2±0.2	0.0±0.0	0.2±0.1	0.1±0.0	0.1±0.1
3	**HGBS**	**SAL**	0.0±0.0	0.0±0.0	0.0±0.0	0.2±0.1	0.0±0.0
3	**LGBS**	**SAL**	0.0±0.0	0.0±0.0	0.1±0.1	0.2±0.0	0.0±0.0
3	**HSIN**	**SAL**	0.0±0.0	0.0±0.0	0.0±0.0	0.4±0.1	0.0±0.0
3	**LSIN**	**SAL**	0.0±0.0	0.0±0.0	0.0±0.0	0.2±0.0	0.1±0.1
9	**HGBS**	**C**	0.0±0.0	0.0±0.0	0.0±0.0	0.2±0.1	0.0±0.0
9	**LGBS**	**C**	0.0±0.0	0.0±0.0	0.1±0.1	0.1±0.0	0.0±0.0
9	**HSIN**	**C**	0.3±0.3	0.0±0.0	0.0±0.0	0.3±0.1	0.0±0.0
9	**LSIN**	**C**	0.0±0.0	0.0±0.0	0.0±0.0	0.2±0.0	0.0±0.0
9	**HGBS**	**CL**	1.8±0.2	0.0±0.0	0.2±0.2	0.2±0.0	0.0±0.0
9	**LGBS**	**CL**	1.5±0.4	0.0±0.0	0.1±0.1	0.3±0.1	0.0±0.0
9	**HSIN**	**CL**	3.8±0.6	0.0±0.0	0.1±0.1	0.2±0.0	0.0±0.0
9	**LSIN**	**CL**	2.2±1.1	0.0±0.0	0.3±0.3	0.2±0.1	0.0±0.0
9	**HGBS**	**JA**	0.2±0.2	0.0±0.0	0.1±0.1	0.2±0.0	0.0±0.0
9	**LGBS**	**JA**	0.0±0.0	0.0±0.0	0.0±0.0	0.3±0.1	0.0±0.0
9	**HSIN**	**JA**	0.0±0.0	0.0±0.0	0.7±0.2	0.3±0.0	0.0±0.0
9	**LSIN**	**JA**	0.2±0.2	0.0±0.0	0.3±0.2	0.3±0.1	0.0±0.0
9	**HGBS**	**JAL**	0.0±0.0	0.0±0.0	0.0±0.0	0.3±0.1	0.0±0.0
9	**LGBS**	**JAL**	0.0±0.0	0.0±0.0	0.0±0.0	0.2±0.0	0.0±0.0
9	**HSIN**	**JAL**	0.0±0.0	0.0±0.0	0.0±0.0	0.1±0.1	0.8±0.2

9	**LSIN**	**JAL**	0.0±0.0	0.0±0.0	0.0±0.0	0.3±0.1	0.0±0.0
9	**HGBS**	**SA**	0.0±0.0	0.0±0.0	0.3±0.2	0.4±0.1	0.0±0.0
9	**LGBS**	**SA**	0.4±0.4	0.0±0.0	0.3±0.3	0.3±0.0	0.0±0.0
9	**HSIN**	**SA**	0.2±0.2	0.0±0.0	0.0±0.0	0.3±0.1	0.0±0.0
9	**LSIN**	**SA**	0.0±0.0	0.0±0.0	0.1±0.1	0.2±0.0	0.0±0.0
9	**HGBS**	**SAL**	0.0±0.0	0.0±0.0	0.0±0.0	0.2±0.0	0.0±0.0
9	**LGBS**	**SAL**	0.0±0.0	0.0±0.0	0.1±0.1	0.2±0.0	0.0±0.0
9	**HSIN**	**SAL**	0.6±0.4	0.0±0.0	0.2±0.1	0.3±0.0	0.0±0.0
9	**LSIN**	**SAL**	0.1±0.1	0.0±0.0	0.1±0.1	0.2±0.0	0.0±0.0

Table S2. Differences in glucosinolate content across times (days after treatment with JA and SA). Test statistic and *P*-values of ANOVA or Kruskall-Wallis test shown to compare differences in glucosinolate content among times (1, 3, and 9 days after treatment in the case of the treatments C, JA, and SA, and 3 and 9 days after treatment in the case of CL, JAL, and SAL) within the same genotype. Significant *P*-values ($P \leq 0.05$) are shown in bold type.

**Treatment**	**Gluc.**	**HGBS**	**LGBS**	**HSIN**	**LSIN**
**C**	**AL**	0.73; *P*=0.495	0.56; *P*=0.581	2.81; *P*=0.082	0.08; *P*=0.924
**C**	**IN**	14.06; ***P*≤0.001**	7.56; ***P*=0.003**	6.07; ***P*=0.008**	1.41; *P*=0.265
**C**	**TO**	2.98; *P*=0.075	2.02; *P*=0.156	6.48; ***P*=0.006**	0.77; *P*=0.476
**C**	**GIB**	1.32; *P*=0.291	0.73; *P*=0.494	2.19; *P*=0.136	0.12; *P*=0.944
**C**	**SIN**	0.46; *P*=0.641	1.26; *P*=0.304	2.11; *P*=0.145	6.81; ***P*=0.033**
**C**	**GBS**	12.15; ***P*≤0.001**	8.04; ***P*=0.002**	6.11; ***P*=0.008**	1.63; *P*=0.218
**C**	**NEO**	11.54; ***P*≤0.001**	1.24; *P*=0.310	5.23; ***P*=0.014**	2.71; *P*=0.258
**JA**	**AL**	9.45; ***P*≤0.001**	1.70; *P*=0.206	12.07; ***P*≤0.001**	1.97; *P*=0.165
**JA**	**IN**	17.57; ***P*≤0.001**	2.28; *P*=0.127	18.48; ***P*≤0.001**	16.67; ***P*≤0.001**
**JA**	**TO**	8.09; ***P*=0.002**	0.66; *P*=0.525	10.33; ***P*≤0.001**	13.89; ***P*≤0.001**
**JA**	**GIB**	4.03; ***P*=0.032**	2.21; *P*=0.134	4.25; ***P*=0.027**	3.20; *P*=0.061
**JA**	**SIN**	8.09; ***P*=0.002**	1.15; *P*=0.336	12.52; ***P*≤0.001**	1.47; *P*=0.253
**JA**	**GBS**	14.75; ***P*≤0.001**	2.42; *P*=0.113	8.76; ***P*=0.002**	7.70; ***P*=0.003**
**JA**	**NEO**	3.25; *P*=0.058	0.38; *P*=0.691	6.24; ***P*=0.007**	5.23; ***P*=0.014**
**SA**	**AL**	16.96; ***P*≤0.001**	3.43; ***P*=0.050**	5.17; ***P*=0.014**	0.43; *P*=0.659
**SA**	**IN**	3.57; ***P*=0.046**	1.42; *P*=0.264	16.00; ***P*≤0.001**	3.44; ***P*=0.050**
**SA**	**TO**	1.08; *P*=0.357	1.35; *P*=0.280	9.66; ***P*≤0.001**	1.51; *P*=0.244
**SA**	**GIB**	10.36; ***P*≤0.001**	4.20; ***P*=0.029**	7.12; ***P*≤0.001**	0.11; *P*=0.900
**SA**	**SIN**	4.62; ***P*=0.022**	1.28; *P*=0.298	1.55; *P*=0.235	1.17; *P*=0.329
**SA**	**GBS**	2.44; *P*=0.112	1.27; *P*=0.301	16.90; ***P*≤0.001**	2.48; *P*=0.107
**SA**	**NEO**	11.56; ***P*≤0.001**	1.46; *P*=0.254	5.52; ***P*=0.011**	6.94; ***P*=0.005**
**CL**	**AL**	40.31; ***P*≤0.001**	23.36; ***P*≤0.001**	0.92; *P*=0.364	1.32; *P*=0.303
**CL**	**IN**	13.19; ***P*=0.008**	3.62; *P*=0.094	0.25; *P*=0.629	3.75; *P*=0.111
**CL**	**TO**	5.09; *P*=0.059	0.37; *P*=0.558	0.02; *P*=0.894	2.67; *P*=0.163

**CL**	**GIB**	0.00; ***P*=0.016**	9.86; ***P*=0.014**	1.05; *P*=0.335	1.58; *P*=0.265
**CL**	**SIN**	17.00; ***P*=0.004**	35.95; ***P*≤0.001**	4.93; *P*=0.057	1.53; *P*=0.272
**CL**	**GBS**	23.41; ***P*=0.002**	2.14; *P*=0.181	0.03; *P*=0.871	6.00; *P*=0.058
**CL**	**NEO**	0.05; *P*=0.828	3.28; *P*=0.108	2.18; *P*=0.178	0.15; *P*=0.713
**JAL**	**AL**	1.88; *P*=0.207	14.36; ***P*=0.005**	8.06; ***P*=0.022**	2.52; *P*=0.164
**JAL**	**IN**	12.47; ***P*=0.008**	1.51; *P*=0.254	4.23; *P*=0.074	4.00; *P*=0.393
**JAL**	**TO**	11.14; ***P*=0.010**	5.53; ***P*=0.046**	9.88; ***P*=0.014**	2.67; *P*=0.153
**JAL**	**GIB**	5.00; *P*=0.056	11.59; ***P*=0.009**	0.08; *P*=0.791	7.88; ***P*=0.031**
**JAL**	**SIN**	0.52; *P*=0.491	3.08; *P*=0.117	6.76; ***P*=0.032**	0.07; *P*=0.797
**JAL**	**GBS**	8.26; ***P*=0.021**	1.29; *P*=0.290	0.08; *P*=0.785	0.45; *P*=0.525
**JAL**	**NEO**	13.03; ***P*=0.007**	0.25; *P*=0.631	2.00; ***P*=0.032**	3.35; *P*=0.117
**SAL**	**AL**	23.95; ***P*≤0.001**	11.49; ***P*=0.009**	6.80; ***P*=0.031**	9.89; ***P*=0.016**
**SAL**	**IN**	0.17; *P*=0.689	0.20; *P*=0.668	0.39; *P*=0.551	2.76; *P*=0.141
**SAL**	**TO**	5.21; *P*=0.052	2.06; *P*=0.189	5.66; ***P*=0.045**	6.42; ***P*=0.039**
**SAL**	**GIB**	6.62; ***P*=0.033**	0.00; ***P*=0.008**	0.00; ***P*=0.008**	13.61; ***P*=0.008**
**SAL**	**SIN**	0.00; ***P*=0.008**	4.46; *P*=0.068	2.99; *P*=0.122	2.22; *P*=0.180
**SAL**	**GBS**	0.23; *P*=0.644	0.13; *P*=0.731	1.51; *P*=0.254	0.14; *P*=0.721
**SAL**	**NEO**	0.05; *P*=0.832	0.57; *P*=0.472	2.81; *P*=0.132	0.88; *P*=0.380

Table S3. Mean ± SE glucosinolate content (μmol g^{-1} plant dry weight) for each treatment and genotype after the application of phytohormones, 1, 3, and 9 days after treatment. The treatments are control (C), jasmonic acid (JA), salicylic acid (SA), control with *M. brassicae* larvae (CL), JA with *M. brassicae* larvae (JAL), and SA with *M. brassicae* larvae (SAL). The genotypes are high in glucobrassicin (HGBS), low in glucobrassicin (LGBS), high in sinigrin (HSIN), and low in sinigrin (LSIN). The glucosinolates shown are glucoiberin (GIB), sinigrin (SIN), glucobrassicin (GBS), neoglucobrassicin (NEO), total aliphatic (AL), total indolic (IN), and total glucosinolates (TO). The less abundant glucosinolates progoitrin (PRO), glucoiberverin (GIV), 4-hydroxyglucobrassicin (OHGBS), 4-methoxyglucobrassicin (MEOHGBS), and gluconasturtiin (GNT) are not shown here, but are shown as supplementary data. For each genotype and treatment, means within a column followed by different letters show significant differences ($P \leq 0.05$) in time (days after treatment). Replication was n=7-10, n=5-10, and n=3-5 for 1, 3, and 9 days after treatment, respectively.

Genotype	Treatment	Days after treatment	GIB	SIN	GBS	NEO	AL	IN	TO
**HGBS**	**C**	**1**	3.9±0.6a	9.3±1.3a	6.8±1.6a	0.3±0.1a	13.3±1.8a	7.5±1.6a	20.8±3.1a
		**3**	4.4±0.6a	10.7±0.9a	9.0±0.4a	0.6±0.2a	15.1±1.3a	9.7±0.5a	24.8±1.5a
		**9**	3.0±0.3a	9.4±1.7a	16.6±2.4b	2.1±0.5b	12.4±1.9a	18.9±2.8b	31.3±4.5a
	**JA**	**1**	6.6±1.2a	13.4±1.6a	20.0±2.8a	8.0±1.7a	20.2±2.4a	29.4±3.5a	50.4±4.5a
		**3**	3.0±0.5b	6.0±1.1b	41.1±3.6b	17.8±4.7a	9.0±1.5b	59.6±5.2b	68.6±5.4b
		**9**	4.7±1.0ab	6.7±2.0b	20.1±2.3a	5.9±1.1a	11.5±1.8b	26.4±2.3a	38.0±3.7a
	**SA**	**1**	7.6±1.0a	12.9±1.7a	8.8±1.4a	0.7±0.2a	21.2±1.5a	10.3±1.4a	31.5±1.8a
		**3**	4.0±0.5b	11.0±1.0a	14.3±2.3a	1.1±0.3a	15.1±1.2b	16.2±2.4b	31.2±3.2a
		**9**	2.5±0.5b	6.3±0.6b	13.6±2.6a	3.0±0.7b	8.8±0.9c	17.2±1.8b	26.1±2.0a
	**CL**	**3**	4.0±0.3a	4.9±0.7a	8.0±1.4a	4.0±1.0a	8.9±0.6a	12.2±2.2a	21.6±2.2a
		**9**	0.9±0.2b	1.8±0.1b	20.6±2.4b	3.7±1.0a	4.5±0.3b	24.7±2.7b	29.2±2.5a
	**JAL**	**3**	4.3±0.7a	4.9±1.5a	60.4±7.8a	22.8±4.3a	9.2±2.2a	83.7±11.0a	92.9±12.4a
		**9**	1.7±0.9a	3.6±0.9a	36.8±2.5b	7.0±0.8b	5.3±1.7a	44.1±2.3b	49.4±3.9b
	**SAL**	**3**	4.3±0.8a	11.4±1.3a	20.7±3.4a	3.4±0.5a	15.7±1.7a	24.3±3.5a	40.0±4.2a
		**9**	2.0±0.4b	3.3±1.1b	18.8±1.9a	3.6±0.9a	5.3±1.3b	22.6±2.1a	27.9±3.2a
**LGBS**	**C**	**1**	4.5±0.7a	7.2±0.7a	4.1±0.5a	0.2±0.1a	12.9±1.2a	4.6±0.5a	16.7±1.4a
		**3**	5.6±0.9a	8.6±1.0a	5.5±0.4a	0.4±0.1a	14.2±1.5a	6.1±0.4a	20.2±1.6a
		**9**	4.0±1.1a	9.6±1.5a	8.5±1.6b	0.3±0.1a	13.6±2.5a	9.0±1.6b	22.7±4.1a
	**JA**	**1**	7.3±1.2a	10.7±2.1a	17.3±2.9a	1.8±0.3a	18.2±3.3a	20.1±3.0a	38.8±4.9a
		**3**	4.1±0.8a	6.9±1.3a	22.1±2.8a	2.4±0.9a	11.3±1.9a	25.0±3.0a	36.4±3.9a
		**9**	6.6±4.1a	9.0±1.9a	12.1±2.6a	2.6±0.8a	15.6±2.7a	15.0±5.2a	30.5±4.3a
	**SA**	**1**	8.3±1.0a	12.8±2.4a	6.1±0.8a	0.7±0.1a	21.5±2.8a	7.0±0.9a	28.5±3.3a
		**3**	6.2±1.0ab	10.0±1.3a	8.6±0.9a	0.4±0.1a	16.4±1.6ab	9.7±1.0a	26.0±2.0a

		**9**	3.8±0.8b	8.2±0.5a	7.4±2.5a	0.7±0.2a	12.4±0.5b	8.6±2.5a	21.0±3.0a
	**CL**	**3**	4.2±0.4a	7.2±0.9a	12.7±1.5a	2.3±0.3a	11.6±1.0a	15.2±1.7a	26.8±1.6a
		**9**	2.4±0.4b	1.6±0.3b	18.4±3.6a	5.6±1.8a	5.5±0.8b	24.4±4.5a	29.9±4.7a
	**JAL**	**3**	5.3±1.0a	6.3±1.6a	35.7±7.3a	4.6±1.0a	11.6±1.2a	40.6±7.5a	52.3±6.7a
		**9**	1.6±0.4b	2.9±1.0a	26.4±3.6a	3.8±1.2a	4.5±1.4b	30.4±3.5a	34.9±3.0b
	**SAL**	**3**	7.5±0.6a	11.4±2.2a	20.8±4.2a	3.1±0.7a	19.0±2.4a	24.2±4.4a	43.2±6.4a
		**9**	3.5±0.5b	6.2±1.1a	18.8±3.7a	2.5±0.5a	9.8±1.3b	21.5±4.2a	31.3±5.3a
**HSIN**	**C**	**1**	3.9±0.5a	12.1±4.2a	6.2±0.8a	0.3±0.1a	16.5±1.6a	7.0±0.9a	23.5±2.3a
		**3**	5.7±0.8a	15.6±1.3a	10.7±1.3b	0.9±0.1ab	21.9±1.9a	11.8±1.4b	33.7±1.9b
		**9**	5.0±0.5a	12.7±1.3a	11.2±1.2b	1.6±0.7b	18.0±1.3a	13.1±1.8b	31.1±2.5b
	**JA**	**1**	4.9±0.7ab	8.6±3.9a	16.8±1.9a	4.6±0.7a	13.7±1.8a	22.0±2.1a	35.7±3.1a
		**3**	3.2±0.5a	7.9±1.3a	40.1±5.9b	13.3±2.7a	11.2±1.5a	54.1±5.5b	65.3±6.1b
		**9**	6.2±0.7b	18.5±2.1b	21.8±2.6a	5.8±1.7a	24.7±2.0b	28.6±2.7a	53.3±4.7b
	**SA**	**1**	4.3±0.5a	13.7±6.4a	5.8±0.7a	0.3±0.1a	18.3±2.0a	6.6±0.8a	24.9±2.5a
		**3**	7.0±0.8b	17.3±1.6a	13.6±1.1b	0.9±0.3ab	24.6±1.5b	15.3±1.3b	39.9±2.4b
		**9**	3.4±0.6a	12.9±1.3a	13.6±2.0b	1.5±0.4b	16.5±1.8a	15.4±2.2b	31.8±3.0ab
	**CL**	**3**	3.0±0.8a	9.0±2.8a	22.8±2.5a	4.9±2.0a	12.2±3.5a	28.1±4.1a	40.7±4.2a
		**9**	2.0±0.4a	2.8±0.4a	22.1±3.7a	8.8±1.7a	8.6±1.3a	31.3±4.9a	39.8±4.5a
	**JAL**	**3**	3.6±1.1a	9.8±2.1a	23.4±6.7a	19.6±6.1a	13.4±2.0a	43.2±3.3a	56.6±2.8a
		**9**	3.1±1.1a	3.4±1.3b	25.8±5.3a	4.6±0.9b	6.5±1.4b	30.5±5.2a	37.7±5.3b
	**SAL**	**3**	6.9±0.8a	13.1±3.1a	20.4±2.8a	13.1±5.8a	20.0±3.6a	34.4±7.7a	54.4±5.0a
		**9**	2.1±0.7b	7.5±0.6a	25.1±2.7a	3.7±1.0a	10.3±1.1b	29.3±2.8a	39.6±3.7b
**LSIN**	**C**	**1**	5.1±1.3a	2.4±0.6a	5.1±0.7a	1.1±0.6a	7.6±1.8a	6.6±1.1a	14.2±2.5a
		**3**	4.0±0.8a	3.5±0.9a	6.9±0.9ab	0.8±0.2a	7.5±1.1a	7.7±0.9a	15.2±1.9a
		**9**	4.3±1.2a	4.1±0.5a	8.5±2.9b	1.7±0.5a	8.4±1.3a	10.4±3.0a	18.9±3.0a
	**JA**	**1**	2.7±0.4a	3.3±1.4a	12.5±1.8a	3.7±1.0a	6.6±1.9a	16.7±2.1a	23.4±3.4a
		**3**	3.5±0.6a	1.8±0.5a	33.0±4.9b	17.2±4.8b	5.4±0.8a	51.0±5.8b	56.3±5.6b
		**9**	5.6±1.5a	4.5±1.3a	20.7±3.8ab	3.6±0.6a	10.3±2.5a	24.9±4.3a	35.2±3.7a
	**SA**	**1**	4.1±0.7a	3.4±0.7a	5.3±0.6a	1.0±0.4a	7.5±1.3a	6.6±0.7a	14.1±1.7a
		**3**	3.7±0.8a	3.8±0.5a	10.4±2.4a	1.1±0.3a	7.6±1.1a	11.8±2.8ab	19.6±3.6a
		**9**	3.8±0.7a	2.2±0.5a	9.3±1.5a	4.8±1.8b	6.0±0.5a	14.4±1.4b	20.4±1.7a
	**CL**	**3**	3.1±0.5a	2.2±0.4a	25.5±4.8a	6.4±1.7a	5.3±0.5a	32.1±5.5a	37.5±6.0a
		**9**	4.3±0.9a	1.3±0.7a	8.8±4.6a	7.8±3.4a	7.8±2.4a	17.0±5.0a	24.8±3.9a
	**JAL**	**3**	4.5±1.0a	1.9±0.5a	21.0±5.1a	24.5±7.1a	6.5±1.2a	45.9±5.5a	52.4±4.9a
		**9**	0.9±0.2b	2.3±1.6a	26.3±5.8a	6.9±2.2a	3.2±1.7a	33.5±8.0a	36.7±9.6a
	**SAL**	**3**	4.1±0.6a	2.9±1.1a	18.9±5.8a	10.8±5.5a	7.1±1.3a	29.9±3.9a	37.1±4.0a
		**9**	1.4±0.3b	1.0±0.2a	16.3±2.4a	4.8±1.5a	2.4±0.3b	21.5±2.8a	23.9±3.0b

Table S4. Effect of JA, SA, CL, JAL, and SAL treatments on glucosinolate content in the different genotypes, 1, 3, and 9 days after treatment with JA and SA. Test statistic and *P*-values of ANOVA or Kruskall-Wallis test shown to compare differences in glucosinolate content among treatments within the same genotype. Treatments included in the comparisons among treatments are C, JA, and SA (also CL for 3 and 9 days after treatment) (A), and JA compared to JAL and SA compared to SAL (3 and 9 days after treatment) (B). Significant *P*-values ($P \leq 0.05$) are shown in bold type.

A

**Comparison**	**Days after treat.**	**Gluc.**	**HGBS**	**LGBS**	**HSIN**	**LSIN**
**C-JA-SA**	**1**	**AL**	7.26; ***P*=0.027**	3.37; ***P*=0.050**	1.69; *P*=0.203	0.50; *P*=0.780
**C-JA-SA**	**1**	**IN**	22.83; ***P*≤0.001**	21.01; ***P*≤0.001**	42.01; ***P*≤0.001**	16.96; ***P*≤0.001**
**C-JA-SA**	**1**	**TO**	18.33; ***P*≤0.001**	9.98; ***P*≤0.001**	6.33; ***P*=0.006**	4.18; ***P*=0.027**
**C-JA-SA**	**1**	**GIB**	3.15; *P*=0.061	3.89; ***P*=0.033**	0.70; *P*=0.507	1.62; *P*=0.218
**C-JA-SA**	**1**	**SIN**	1.66; *P*=0.212	4.54; *P*=0.103	2.84; *P*=0.076	1.66; *P*=0.437
**C-JA-SA**	**1**	**GBS**	13.08; ***P*≤0.001**	16.49; ***P*≤0.001**	24.46; ***P*≤0.001**	13.28; ***P*≤0.001**
**C-JA-SA**	**1**	**NEO**	15.73; ***P*≤0.001**	14.79; ***P*≤0.001**	35.96; ***P*≤0.001**	1.66; ***P*=0.016**
**C-JA-SA-CL**	**3**	**AL**	6.67; ***P*≤0.001**	2.02; *P*=0.132	12.86; ***P*≤0.001**	1.37; *P*=0.272
**C-JA-SA-CL**	**3**	**IN**	52.29; ***P*≤0.001**	23.90; ***P*≤0.001**	33.32; ***P*≤0.001**	27.78; ***P*≤0.001**
**C-JA-SA-CL**	**3**	**TO**	34.72; ***P*≤0.001**	7.28; ***P*≤0.001**	12.90; ***P*≤0.001**	21.86; ***P*≤0.001**
**C-JA-SA-CL**	**3**	**GIB**	1.36; *P*=0.275	1.30; *P*=0.294	6.86; ***P*≤0.001**	0.20; *P*=0.272
**C-JA-SA-CL**	**3**	**SIN**	8.77; ***P*≤0.001**	1.39; *P*=0.266	8.83; ***P*≤0.001**	2.20; *P*=0.109
**C-JA-SA-CL**	**3**	**GBS**	41.01; ***P*≤0.001**	20.86; ***P*≤0.001**	15.26; ***P*≤0.001**	13.85; ***P*≤0.001**
**C-JA-SA-CL**	**3**	**NEO**	27.39; ***P*≤0.001**	5.15; ***P*=0.005**	14.54; ***P*≤0.001**	24.65; ***P*≤0.001**
**C-JA-SA-CL**	**9**	**AL**	5.42; ***P*=0.010**	5.24; ***P*=0.010**	16.49; ***P*≤0.001**	1.11; *P*=0.377
**C-JA-SA-CL**	**9**	**IN**	3.35; ***P*=0.047**	6.20; ***P*=0.005**	8.56; ***P*≤0.001**	8.16; ***P*=0.043**
**C-JA-SA-CL**	**9**	**TO**	2.26; *P*=0.124	1.44; *P*=0.268	7.38; ***P*=0.003**	6.31; ***P*=0.006**
**C-JA-SA-CL**	**9**	**GIB**	5.64; ***P*=0.009**	2.27; *P*=0.120	9.85; ***P*≤0.001**	0.43; *P*=0.735
**C-JA-SA-CL**	**9**	**SIN**	4.60; ***P*=0.018**	8.73; ***P*≤0.001**	21.60; ***P*≤0.001**	7.87; ***P*=0.049**
**C-JA-SA-CL**	**9**	**GBS**	1.83; *P*=0.186	3.44; ***P*=0.042**	4.92; ***P*=0.013**	6.39; *P*=0.094
**C-JA-SA-CL**	**9**	**NEO**	3.92; ***P*=0.030**	5.96; ***P*=0.006**	12.76; ***P*=0.005**	2.33; *P*=0.119

B

**Comparison**	**Days after treat.**	**Gluc.**	**HGBS**	**LGBS**	**HSIN**	**LSIN**
**JA-JAL**	**3**	**AL**	0.01; *P*=0.947	0.01; *P*=0.913	0.69; *P*=0421	0.57; *P*=0.464
**JA-JAL**	**3**	**IN**	5.17; ***P*=0.041**	5.25; ***P*=0.041**	1.73; *P*=0.211	0.30; *P*=0.591
**JA-JAL**	**3**	**TO**	4.43; *P*=0.055	4.91; ***P*=0.047**	19.00; *P*=0.513	0.21; *P*=0.657
**JA-JAL**	**3**	**GIB**	2.13; *P*=0.168	0.92; *P*=0.355	0.09; *P*=0.769	0.97; *P*=0.342
**JA-JAL**	**3**	**SIN**	0.36; *P*=0.561	0.10; *P*=0.752	0.72; *P*=0.411	0.05; *P*=0.827
**JA-JAL**	**3**	**GBS**	43.00; ***P*=0.028**	4.33; *P*=0.059	2.98; *P*=0.108	2.33; *P*=0.151
**JA-JAL**	**3**	**NEO**	36.00; *P*=0.206	2.31; *P*=0.154	1.23; *P*=0.287	0.76; *P*=0.399
**JA-JAL**	**9**	**AL**	6.17; ***P*=0.038**	13.02; ***P*=0.007**	54.35; ***P*≤0.001**	4.02; *P*=0.092
**JA-JAL**	**9**	**IN**	25.00; ***P*=0.008**	13.46; ***P*=0.006**	0.10; *P*=0.761	13.00; *P*=0.143
**JA-JAL**	**9**	**TO**	19.00; *P*=0.222	0.70; *P*=0.427	4.86; *P*=0.059	0.03; *P*=0.870
**JA-JAL**	**9**	**GIB**	4.57; *P*=0.065	7.04; ***P*=0.029**	5.54; ***P*=0.046**	5.20; *P*=0.063
**JA-JAL**	**9**	**SIN**	2.00; *P*=0.195	8.02; ***P*=0.022**	36.16; ***P*≤0.001**	1.12; *P*=0.331
**JA-JAL**	**9**	**GBS**	23.90; ***P*≤0.001**	10.31; ***P*=0.012**	0.45; *P*=0.521	13.00; *P*=0.143
**JA-JAL**	**9**	**NEO**	0.63; *P*=0.452	0.64; *P*=0.448	0.39; *P*=0.548	3.41; *P*=0.114
**SA-SAL**	**3**	**AL**	0.09; *P*=0.773	0.83; *P*=0.378	1.99; *P*=0.182	0.09; *P*=0.766
**SA-SAL**	**3**	**IN**	3.79; *P*=0.075	19.42; ***P*≤0.001**	11.88; ***P*=0.004**	14.16; ***P*=0.002**
**SA-SAL**	**3**	**TO**	2.74; *P*=0.124	44.00; ***P*=0.019**	8.80; ***P*=0.011**	9.19; ***P*=0.010**
**SA-SAL**	**3**	**GIB**	0.10; *P*=0.753	0.82; *P*=0.382	0.01; *P*=0.933	0.14; *P*=0.718
**SA-SAL**	**3**	**SIN**	22.00; *P*=1.000	0.37; *P*=0.553	1.75; *P*=0.208	0.54; *P*=0.476
**SA-SAL**	**3**	**GBS**	34.00; *P*=0.147	14.79; ***P*=0.002**	7.49; ***P*=0.017**	2.58; *P*=0.132
**SA-SAL**	**3**	**NEO**	43.00; ***P*=0.004**	30.79; ***P*≤0.001**	10.20; ***P*=0.007**	6.64; ***P*=0.023**
**SA-SAL**	**9**	**AL**	4.87; *P*=0.058	3.81; *P*=0.087	8.48; ***P*=0.020**	34.11; ***P*≤0.001**
**SA-SAL**	**9**	**IN**	3.73; *P*=0.090	6.79; ***P*=0.031**	14.99; ***P*=0.005**	5.60; ***P*=0.050**
**SA-SAL**	**9**	**TO**	0.24; *P*=0.636	2.82; *P*=0.132	2.66; *P*=0.141	1.12; *P*=0.326
**SA-SAL**	**9**	**GIB**	0.57; *P*=0.471	12.00; *P*=1.000	6.00; *P*=0.222	**7.43; *P*=0.030**
**SA-SAL**	**9**	**SIN**	5.70; ***P*=0.044**	2.88; *P*=0.128	14.44; ***P*=0.005**	1.00; ***P*=0.032**

**SA-SAL**	**9**	**GBS**	2.63; *P*=0.144	6.33; ***P*=0.036**	11.79; ***P*=0.009**	6.45; ***P*=0.039**
**SA-SAL**	**9**	**NEO**	0.27; *P*=0.619	10.75; ***P*=0.011**	3.96; *P*=0.082	0.00; *P*=0.981

Table S5. Mean ± SE glucosinolate content (µmol g^{-1} plant dry weight) for each treatment and genotype after the application of phytohormones. The treatments are control (C), jasmonic acid (JA), salicylic acid (SA), and control with *M. brassicae* larvae (CL). The genotypes are high in glucobrassicin (HGBS), low in glucobrassicin (LGBS), high in sinigrin (HSIN), and low in sinigrin (LSIN). The glucosinolates shown are glucoiberin (GIB), sinigrin (SIN), glucobrassicin (GBS), neoglucobrassicin (NEO), total aliphatic (AL), total indolic (IN), and total glucosinolates (TO). The less abundant glucosinolates progoitrin (PRO), glucoiberverin (GIV), 4-hydroxyglucobrassicin (OHGBS), 4-methoxyglucobrassicin (MEOHGBS), and gluconasturtiin (GNT) are not shown here, but are shown as supplementary data. For each time (days after treatment) and genotype, means within a column followed by different letters show significant treatment differences ($P \leq 0.05$) among genotypes. Replication was n=7-10, n=5-10, and n=3-5 for 1, 3, and 9 days after treatment, respectively.

Days after treatment	Genotype	Treatment	GIB	SIN	GBS	NEO	AL	IN	TO
1	HGBS	C	3.9±0.6a	9.3±1.3a	6.8±1.6a	0.3±0.1a	13.3±1.8a	7.5±1.6a	20.8±3.1a
		JA	6.6±1.2a	13.4±1.6a	20.0±2.8b	8.0±1.7b	20.2±2.4ab	29.4±3.5b	50.4±4.5b
		SA	7.6±1.0a	12.9±1.7a	8.8±1.4a	0.7±0.2a	21.2±1.5b	10.3±1.4a	31.5±1.8c
	LGBS	C	4.5±0.7a	7.2±0.7a	4.1±0.5a	0.2±0.1a	12.9±1.2a	4.6±0.5a	16.7±1.4a
		JA	7.3±1.2ab	10.7±2.1a	17.3±2.9b	1.8±0.3b	18.2±3.3ab	20.1±3.0b	38.8±4.9b
		SA	8.3±1.0b	12.8±2.4a	6.1±0.8a	0.7±0.1c	21.5±2.8b	7.0±0.9a	28.5±3.3c
	HSIN	C	3.9±0.5a	12.1±4.2a	6.2±0.8a	0.3±0.1a	16.5±1.6a	7.0±0.9a	23.5±2.3a
		JA	4.9±0.7a	8.6±3.9a	16.8±1.9b	4.6±0.7b	13.7±1.8a	22.0±2.1b	35.7±3.1b
		SA	4.3±0.5a	13.7±6.4a	5.8±0.7a	0.3±0.1a	18.3±2.0a	6.6±0.8a	24.9±2.5a
	LSIN	C	5.1±1.3a	2.4±0.6a	5.1±0.7a	1.1±0.6a	7.6±1.8a	6.6±1.1a	14.2±2.5a
		JA	2.7±0.4a	3.3±1.4a	12.5±1.8b	3.7±1.0b	6.6±1.9a	16.7±2.1b	23.4±3.4b
		SA	4.1±0.7a	3.4±0.7a	5.3±0.6a	1.0±0.4a	7.5±1.3a	6.6±0.7a	14.1±1.7a
3	HGBS	C	4.4±0.6a	10.7±0.9a	9.0±0.4a	0.6±0.2a	15.1±1.3a	9.7±0.5a	24.8±1.5a
		JA	3.0±0.5a	6.0±1.1b	41.1±3.6b	17.8±4.7b	9.0±1.5b	59.6±5.2b	68.6±5.4b
		SA	4.0±0.5a	11.0±1.0a	14.3±2.3a	1.1±0.3a	15.1±1.2a	16.2±2.4a	31.2±3.2a
		CL	4.0±0.3a	4.9±0.7b	8.0±1.4a	4.0±1.0b	8.9±0.6b	12.2±2.2a	21.6±2.2a
	LGBS	C	5.6±0.9a	8.6±1.0a	5.5±0.4a	0.4±0.1a	14.2±1.5a	6.1±0.4a	20.2±1.6a
		JA	4.1±0.8a	6.9±1.3a	22.1±2.8b	2.4±0.9b	11.3±1.9a	25.0±3.0b	36.4±3.9b
		SA	6.2±1.0a	10.0±1.3a	8.6±0.9ac	0.4±0.1a	16.4±1.6a	9.7±1.0c	26.0±2.0a
		CL	4.2±0.4a	7.2±0.9a	12.7±1.5bc	2.3±0.3b	11.6±1.0a	15.2±1.7bc	26.8±1.6a
	HSIN	C	5.7±0.8a	15.6±1.3a	10.7±1.3a	0.9±0.1a	21.9±1.9a	11.8±1.4a	33.7±1.9a
		JA	3.2±0.5b	7.9±1.3b	40.1±5.9b	13.3±2.7b	11.2±1.5b	54.1±5.5b	65.3±6.1b
		SA	7.0±0.8a	17.3±1.6a	13.6±1.1ac	0.9±0.3a	24.6±1.5a	15.3±1.3a	39.9±2.4a
		CL	3.0±0.8b	9.0±2.8b	22.8±2.5c	4.9±2.0ab	12.2±3.5b	28.1±4.1a	40.7±4.2a

	**LSIN**	**C**	4.0±0.8a	3.5±0.9a	6.9±0.9a	0.8±0.2a	7.5±1.1a	7.7±0.9a	15.2±1.9a
		**JA**	3.5±0.6a	1.8±0.5a	33.0±4.9b	17.2±4.8b	5.4±0.8a	51.0±5.8b	56.3±5.6b
		**SA**	3.7±0.8a	3.8±0.5a	10.4±2.4a	1.1±0.3a	7.6±1.1a	11.8±2.8a	19.6±3.6a
		**CL**	3.1±0.5a	2.2±0.4a	25.5±4.8b	6.4±1.7ab	5.3±0.5a	32.1±5.5ab	37.5±6.0c
**9**	**HGBS**	**C**	3.0±0.3ab	9.4±1.7a	16.6±2.4a	2.1±0.5a	12.4±1.9a	18.9±2.8a	31.3±4.5a
		**JA**	4.7±1.0a	6.7±2.0a	20.1±2.3a	5.9±1.1b	11.5±1.8a	26.4±2.3b	38.0±3.7a
		**SA**	2.5±0.5bc	6.3±0.6a	13.6±2.6a	3.0±0.7a	8.8±0.9ab	17.2±1.8a	26.1±2.0a
		**CL**	0.9±0.2c	1.8±0.1b	20.6±2.4a	3.7±1.0ab	4.5±0.3b	24.7±2.7ab	29.2±2.5a
	**LGBS**	**C**	4.0±1.1a	9.6±1.5a	8.5±1.6a	0.3±0.1a	13.6±2.5a	9.0±1.6a	22.7±4.1a
		**JA**	6.6±4.1a	9.0±1.9a	12.1±2.6ab	2.6±0.8a	15.6±2.7a	15.0±2.3a	30.5±4.3a
		**SA**	3.8±0.8a	8.2±0.5a	7.4±2.5a	0.7±0.2a	12.4±0.5a	8.6±2.5a	21.0±3.0a
		**CL**	2.4±0.4a	1.6±0.3b	18.4±3.6b	5.6±1.8b	5.5±0.8b	24.4±4.5b	29.9±4.7a
	**HSIN**	**C**	5.0±0.5abc	12.7±1.3a	11.2±1.2a	1.6±0.7a	18.0±1.3a	13.1±1.8a	31.1±2.5a
		**JA**	6.2±0.7b	18.5±2.1b	21.8±2.6b	5.8±1.7b	24.7±2.0b	28.6±2.7b	53.3±4.7b
		**SA**	3.4±0.6c	12.9±1.3a	13.6±2.0a	1.5±0.4a	16.5±1.8a	15.4±2.2a	31.8±3.0a
		**CL**	2.0±0.4cd	2.8±0.4c	22.1±3.7b	8.8±1.7b	8.6±1.3c	31.3±4.9b	39.8±4.5a
	**LSIN**	**C**	4.3±1.2a	4.1±0.5a	8.5±2.9a	1.7±0.5a	8.4±1.3a	10.4±3.0a	18.9±3.0a
		**JA**	5.6±1.5a	4.5±1.3a	20.7±3.8a	3.6±0.6a	10.3±2.5a	24.9±4.3b	35.2±3.7b
		**SA**	3.8±0.7a	2.2±0.5ab	9.3±1.5a	4.8±1.8a	6.0±0.5a	14.4±1.4a	20.4±1.7a
		**CL**	4.3±0.9a	1.3±0.7b	8.8±4.6a	7.8±3.4a	7.8±2.4a	17.0±5.0ab	24.8±3.9a

Table S6. Mean ± SE glucosinolate content (μmol g^{-1} plant dry weight) in kale genotypes after the application of phytohormones to genotypes high in glucobrassicin (HGBS), low in glucobrassicin (LGBS), high in sinigrin (HSIN), and low in sinigrin (LSIN) (n=3-10). The treatments are jasmonic acid (JA), salicylic acid (SA), JA with *M. brassicae* larvae (JAL), and SA with *M. brassicae* larvae (SAL). The glucosinolates shown glucoiberin (GIB), sinigrin (SIN), glucobrassicin (GBS), neoglucobrassicin (NEO), total aliphatic (AL), total indolic (IN), and total glucosinolates (TO). The less abundant glucosinolates progoitrin (PRO), glucoiberverin (GIV), 4-hydroxyglucobrassicin (OHGBS), 4-methoxyglucobrassicin (MEOHGBS), and gluconasturtiin (GNT) are shown as supplementary data. For each time (days after treatment) and genotype, means within a column followed by different letters show significant treatment differences ($P \leq 0.05$) among genotypes. Replication was n=7-10, n=5-10, and n=3-5 for 1, 3, and 9 days after treatment, respectively.

Days after treatment	Genotype	Treatment	GIB	SIN	GBS	NEO	AL	IN	TO
**3**	**HGBS**	**JA**	3.0±0.5a	6.0±1.1a	41.1±3.6a	17.8±4.7a	9.0±1.5a	59.6±5.2a	68.6±5.4a
		**JAL**	4.3±0.7a	4.9±1.5a	60.4±7.8b	22.8.2±4.3a	9.2±2.2a	83.7±11.0b	92.9±12.4a
		**SA**	4.0±0.5a	11.0±1.0a	14.3±2.3a	1.1±0.3a	15.1±1.2a	16.2±2.4a	31.2±3.2a
		**SAL**	4.3±0.8a	11.4±1.3a	20.7±3.4a	3.4±0.5b	15.7±1.7a	24.3±3.5a	40.0±4.2a
	**LGBS**	**JA**	4.1±0.8a	6.9±1.3a	22.1±2.8a	2.4±0.9a	11.3±1.9a	25.0±3.0a	36.4±3.9a
		**JAL**	5.3±1.0a	6.3±1.6a	35.7±7.3a	4.6±1.0a	11.6±1.2a	40.6±7.5b	52.3±6.7b
		**SA**	6.2±1.0a	10.0±1.3a	8.6±0.9a	0.4±0.1a	16.4±1.6a	9.7±1.0a	26.0±2.0a
		**SAL**	7.5±0.6a	11.4±2.2a	20.8±4.2b	3.1±0.7b	19.0±2.4a	24.2±4.4b	43.2±6.4b
	**HSIN**	**JA**	3.2±0.5a	7.9±1.3a	40.1±5.9a	13.3±2.7a	11.2±1.5a	54.1±5.5a	65.3±6.1a
		**JAL**	3.6±1.1a	9.8±2.1a	23.4±6.7a	19.6±6.1a	13.4±2.0a	43.2±3.3a	56.6±2.8a
		**SA**	7.0±0.8a	17.3±1.6a	13.6±1.1a	0.9±0.3a	24.6±1.5a	15.2±1.3a	39.9±2.4a
		**SAL**	6.9±0.8a	13.1±3.1a	20.4±2.8b	13.1±5.8b	20.0±3.6a	34.4±7.7b	54.4±5.0b
	**LSIN**	**JA**	3.5±0.6a	1.8±0.5a	33.0±4.9a	17.2±4.8a	5.4±0.8a	51.0±5.8a	56.3±5.6a
		**JAL**	4.5±1.0a	1.9±0.5a	21.0±5.1a	24.5±7.1a	6.5±1.2a	45.9±5.5a	52.4±4.9a
		**SA**	3.7±0.8a	3.8±0.5a	10.4±2.4a	1.1±0.3a	7.6±1.1a	11.8±2.8a	19.6±3.6a
		**SAL**	4.1±0.6a	2.9±1.1a	18.9±5.8a	10.8±5.5b	7.1±1.3a	29.9±3.9b	37.1±4.0b
**9**	**HGBS**	**JA**	4.7±1.0a	6.7±2.0a	20.1±2.3a	5.9±1.1a	11.5±1.8a	26.4±2.3a	38.0±3.7a
		**JAL**	1.7±0.9a	3.6±0.9a	36.8±2.5b	7.0±0.8a	5.3±1.7b	44.1±2.3b	49.4±3.9a
		**SA**	2.5±0.5a	6.3±0.6a	13.6±2.6a	3.0±0.7a	8.8±0.9a	17.3±1.8a	26.1±2.0a
		**SAL**	2.0±0.4a	3.3±1.1b	18.8±1.9a	3.6±0.9a	5.3±1.3a	22.6±2.1a	27.9±3.2a
	**LGBS**	**JA**	6.6±1.8a	9.0±1.9a	12.1±2.6a	2.6±0.8a	15.6±2.7a	15.0±2.3a	30.5±4.3a
		**JAL**	1.6±0.4b	2.9±1.0b	26.4±3.6b	3.8±1.2a	4.5±1.4b	30.4±3.5b	34.9±3.0a
		**SA**	3.8±0.8a	8.2±0.5a	7.4±2.5a	0.7±0.2a	12.4±0.5a	8.6±2.5a	21.0±3.0a

	**SAL**	3.5±0.5a	6.2±1.1a	18.8±3.7a	2.5±0.5a	9.8±1.3a	21.5±4.2b	31.3±5.3a
**HSIN**	**JA**	6.2±0.7a	18.5±2.1a	21.8±2.6a	5.8±1.7a	24.7±2.0a	28.6±2.7a	53.3±4.7a
	**JAL**	3.1±1.1b	3.4±1.3b	25.8±5.3a	4.6±0.9a	6.5±1.4b	30.5±5.2a	37.7±5.3a
	**SA**	3.4±0.6a	12.9±1.3a	13.6±2.0a	1.5±0.4a	16.5±1.8a	15.4±2.2a	31.8±3.0a
	**SAL**	2.1±0.7a	7.5±0.6b	25.1±2.7b	3.7±1.0a	10.3±1.1b	29.3±2.8b	39.6±3.7a
**LSIN**	**JA**	5.6±1.5a	4.5±1.3a	20.7±3.8a	3.6±0.6a	10.3±2.5a	24.9±4.3a	35.2±3.7a
	**JAL**	0.9±0.2a	2.3±1.6a	26.3±5.8a	6.9±2.2a	3.2±1.7a	33.5±8.0a	36.7±9.6a
	**SA**	3.8±0.7a	2.2±0.5a	9.3±1.5a	4.8±1.8a	6.0±0.5a	14.4±1.4a	20.4±1.7a
	**SAL**	1.4±0.3b	1.0±0.2a	16.3±2.4b	4.8±1.5a	2.4±0.3b	21.5±2.8b	23.9±3.0a

Table S7. Changes in glucosinolate content among genotypes under C, JA, SA, CL, JAL treatments 1, 3, and 9 days after treatment with JA and SA. Test statistic and *P*-values of ANOVA or Kruskall-Wallis test shown to compare differences in glucosinolate content among genotypes subject to the same treatment. Significant *P*-values ($P \leq 0.05$) are shown in bold type.

**Days after treat.**	**Gluc.**	**C**	**JA**	**SA**	**CL**	**JAL**	**SAL**
**1**	**AL**	11.06; ***P*=0.011**	5.87; ***P*=0.002**	10.82; ***P*≤0.001**	**-**	**-**	**-**
**1**	**IN**	4.89; *P*=0.180	3.75; ***P*=0.019**	3.29; ***P*=0.031**	**-**	**-**	**-**
**1**	**TO**	3.43; ***P*=0.028**	7.11; ***P*≤0.001**	9.95; ***P*≤0.001**	**-**	**-**	**-**
**1**	**GIB**	0.39; *P*=0.764	4.39; ***P*=0.010**	7.27; ***P*≤0.001**	**-**	**-**	**-**
**1**	**SIN**	23.69; ***P*≤0.001**	16.05; ***P*≤0.001**	21.68; ***P*≤0.001**	**-**	**-**	**-**
**1**	**GBS**	4.72; *P*=0.194	1.58; *P*=0.212	2.62; *P*=0.066	**-**	**-**	**-**
**1**	**NEO**	9.22; ***P*=0.027**	5.96; ***P*=0.002**	1.79; *P*=0.167	**-**	**-**	**-**
**3**	**AL**	15.69; ***P*≤0.001**	3.73; ***P*=0.020**	25.18; ***P*≤0.001**	2.32; *P*=0.116	3.05; *P*=0.059	5.99; ***P*=0.006**
**3**	**IN**	7.47; ***P*≤0.001**	8.57; ***P*≤0.001**	2.25; *P*=0.100	7.56; ***P*=0.003**	7.78; ***P*=0.002**	0.91; *P*=0.457
**3**	**TO**	20.29; ***P*≤0.001**	6.86; ***P*≤0.001**	9.27; ***P*≤0.001**	6.28; ***P*=0.006**	6.70; ***P*=0.004**	2.30; *P*=0.116
**3**	**GIB**	1.14; *P*=0.345	0.60; *P*=0.618	4.16; ***P*=0.013**	1.36; *P*=0.293	0.62; *P*=0.610	6.34; ***P*=0.005**
**3**	**SIN**	23.53; ***P*≤0.001**	6.41; ***P*≤0.001**	22.34; ***P*≤0.001**	3.19; *P*=0.055	4.51; ***P*=0.018**	4.70; ***P*=0.015**
**3**	**GBS**	7.11; ***P*≤0.001**	3.54; ***P*=0.024**	2.21; *P*=0.104	9.94; ***P*≤0.001**	7.04; ***P*=0.003**	0.04; *P*=0.988
**3**	**NEO**	7.56; *P*=0.056	15.49; ***P*≤0.001**	1.77; *P*=0.170	1.46; *P*=0.264	3.05; *P*=0.059	1.74; *P*=0.200
**9**	**AL**	4.69; ***P*=0.016**	8.08; ***P*=0.002**	17.79; ***P*≤0.001**	2.54; *P*=0.102	0.67; *P*=0.377	10.41; ***P*≤0.001**
**9**	**IN**	3.36; ***P*=0.045**	3.97; ***P*=0.027**	3.28; ***P*=0.048**	1.50; *P*=0.260	6.61; *P*=0.085	1.47; *P*=0.263
**9**	**TO**	2.99; *P*=0.062	5.71; ***P*=0.007**	4.54; ***P*=0.017**	2.20; *P*=0.137	1.84; *P*=0.187	2.69; *P*=0.084
**9**	**GIB**	0.87; *P*=0.478	0.39; *P*=0.760	0.82; *P*=0.499	9.50; ***P*=0.023**	1.19; *P*=0.349	7.66; *P*=0.054
**9**	**SIN**	7.27; ***P*=0.003**	10.99; ***P*≤0.001**	31.35; ***P*≤0.001**	2.78; *P*=0.083	0.20; *P*=0.895	10.36; ***P*≤0.001**
**9**	**GBS**	3.24; ***P*=0.050**	2.40; *P*=0.106	2.00; *P*=0.154	2.18; *P*=0.139	1.67; *P*=0.219	1.76; *P*=0.197
**9**	**NEO**	9.83; ***P*=0.020**	2.08; *P*=0.144	3.22; *P*=0.051	1.44; *P*=0.276	1.90; *P*=0.176	0.90; *P*=0.465

Table S8. Mean ± SE glucosinolate content (µmol g^{-1} plant dry weight) for each treatment and genotype after the application of phytohormones (n=3-10). The treatments are control (C), jasmonic acid (JA), salicylic acid (SA), control with *M. brassicae* larvae (CL), JA with *M. brassicae* larvae (JAL), and SA with *M. brassicae* larvae (SAL). The genotypes are high in glucobrassicin (HGBS), low in glucobrassicin (LGBS), high in sinigrin (HSIN), and low in sinigrin (LSIN). The glucosinolates shown are glucoiberin (GIB), sinigrin (SIN), glucobrassicin (GBS), neoglucobrassicin (NEO), total aliphatic (AL), total indolic (IN), and total glucosinolates (TO). The less abundant glucosinolates progoitrin (PRO), glucoiberverin (GIV), 4-hydroxyglucobrassicin (OHGBS), 4-methoxyglucobrassicin (MEOHGBS), and gluconasturtiin (GNT) are not shown here, but are shown as supplementary data. For each time (days after treatment) and treatment, means within a column followed by different letters show significant differences ($P \leq 0.05$) among genotypes. Replication was n=7-10, n=5-10, and n=3-5 for 1, 3, and 9 days after treatment, respectively.

Days after treatment	Treatment	Genotype	GIB	SIN	GBS	NEO	AL	IN	TO
1	**C**	**HGBS**	3.9±0.6a	9.3±1.3a	6.8±1.6a	0.3±0.1ab	13.3±1.8ab	7.5±1.6a	20.8±3.1ab
		**LGBS**	4.5±0.7a	7.2±0.7ab	4.1±0.5a	0.2±0.1a	12.9±1.2ab	4.6±0.5a	16.7±1.4a
		**HSIN**	3.9±0.5a	12.1±4.2a	6.2±0.8a	0.3±0.1ab	16.5±1.6a	7.0±0.9a	23.5±2.3b
		**LSIN**	5.1±1.3a	2.4±0.6b	5.1±0.7a	1.1±0.6b	7.6±1.8b	6.6±1.1a	14.2±2.5a
	**JA**	**HGBS**	6.6±1.2a	13.4±1.6a	20.0±2.8a	8.0±1.7a	20.2±2.4a	29.4±3.5a	50.4±4.5a
		**LGBS**	7.3±1.2a	10.7±2.1a	17.3±2.9a	1.8±0.3b	18.2±3.3a	20.1±3.0b	38.8±4.9ab
		**HSIN**	4.9±0.7ab	8.6±3.9ab	16.8±1.9a	4.6±0.7b	13.7±1.8ab	22.0±2.1b	35.7±3.1b
		**LSIN**	2.7±0.4b	3.3±1.4b	12.5±1.8a	3.7±1.0b	6.6±1.9b	16.7±2.1b	23.4±3.4c
	**SA**	**HGBS**	7.6±1.0a	12.9±1.7a	8.8±1.4b	0.7±0.2a	21.2±1.5a	10.3±1.4a	31.5±1.8a
		**LGBS**	8.3±1.0a	12.8±2.4a	6.1±0.8a	0.7±0.1a	21.5±2.8a	7.0±0.9b	28.5±3.3a
		**HSIN**	4.3±0.5b	13.7±6.4a	5.8±0.7a	0.3±0.1a	18.3±2.0a	6.6±0.8b	24.9±2.5a
		**LSIN**	4.1±0.7b	3.4±0.7b	5.3±0.6a	1.0±0.4a	7.5±1.3b	6.6±0.7b	14.1±1.7b
3	**C**	**HGBS**	4.4±0.6a	10.7±0.9a	9.0±0.4a	0.6±0.2a	15.1±1.3a	9.7±0.5a	24.8±1.5a
		**LGBS**	5.6±0.9a	8.6±1.0a	5.5±0.4b	0.4±0.1a	14.2±1.5a	6.1±0.4b	20.2±1.6a
		**HSIN**	5.7±0.8a	15.6±1.3b	10.7±1.3a	0.9±0.1a	21.9±1.9b	11.8±1.4a	33.7±1.9b
		**LSIN**	4.0±0.8a	3.5±0.9c	6.9±0.9b	0.8±0.2a	7.5±1.1c	7.7±0.9ab	15.2±1.9c
	**JA**	**HGBS**	3.0±0.5a	6.0±1.1a	41.1±3.6a	17.8±4.7a	9.0±1.5ab	59.6±5.2a	68.6±5.4a
		**LGBS**	4.1±0.8a	6.9±1.3a	22.1±2.8b	2.4±0.9b	11.3±1.9a	25.0±3.0b	36.4±3.9b
		**HSIN**	3.2±0.5a	7.9±1.3a	40.1±5.9a	13.3±2.7a	11.2±1.5a	54.1±5.5a	65.3±6.1a
		**LSIN**	3.5±0.6a	1.8±0.5b	33.0±4.9ab	17.2±4.8a	5.4±0.8b	51.0±5.8a	56.3±5.6a
	**SA**	**HGBS**	4.0±0.5ab	11.0±1.0a	14.3±2.3a	1.1±0.3a	15.1±1.2a	16.2±2.4a	31.2±3.2a

		**LGBS**	6.2±1.0b	10.0±1.3a	8.6±0.9a	0.4±0.1a	16.4±1.6a	9.7±1.0a	26.0±2.0ac
		**HSIN**	7.0±0.8c	17.3±1.6b	13.6±1.1a	0.9±0.3a	24.6±1.5b	15.3±1.3a	39.9±2.4b
		**LSIN**	3.7±0.8a	3.8±0.5c	10.4±2.4a	1.1±0.3a	7.6±1.1c	11.8±2.8a	19.6±3.6c
	**CL**	**HGBS**	4.0±0.3a	4.9±0.7a	8.0±1.4a	4.0±1.0a	8.9±0.6a	12.2±2.2a	21.6±2.2a
		**LGBS**	4.2±0.4a	7.2±0.9a	12.7±1.5a	2.3±0.3a	11.6±1.0a	15.2±1.7a	26.8±1.6ac
		**HSIN**	3.0±0.8a	9.0±2.8a	22.8±2.5b	4.9±2.0a	12.2±3.5a	28.1±4.1b	40.7±4.2b
		**LSIN**	3.1±0.5a	2.2±0.4a	25.5±4.8b	6.4±1.7a	5.3±0.5a	32.1±5.5b	37.5±6.0bc
	**JAL**	**HGBS**	4.3±0.7a	4.9±1.5a	60.4±7.8a	22.8±4.3a	9.2±2.2a	83.7±11.0a	92.9±12.4a
		**LGBS**	5.3±1.0a	6.3±1.6ab	35.7±7.3b	4.6±1.0a	11.6±1.2a	40.6±7.5b	52.3±6.7b
		**HSIN**	3.6±1.1a	9.8±2.1b	23.4±6.7b	19.6±6.1a	13.4±2.0a	43.2±3.3b	56.6±2.8b
		**LSIN**	4.5±1.0a	1.9±0.5a	21.0±5.1b	24.5±7.1a	6.5±1.2a	45.9±5.5b	52.4±4.9b
	**SAL**	**HGBS**	4.3±0.8a	11.4±1.3a	20.7±3.4a	3.4±0.5a	15.7±1.7a	24.3±3.5a	40.0±4.2a
		**LGBS**	7.5±0.6b	11.4±2.2a	20.8±4.2a	3.1±0.7a	19.0±2.4a	24.2±4.4a	43.2±6.4ac
		**HSIN**	6.9±0.8b	13.1±3.1a	20.4±2.8a	13.1±5.8a	20.0±3.6a	34.4±7.7a	54.4±5.0a
		**LSIN**	4.1±0.6a	2.9±1.1b	18.9±5.8a	10.8±5.5a	7.1±1.3b	29.9±3.9a	37.1±4.0a
**9**	**C**	**HGBS**	3.0±0.3a	9.4±1.7a	16.6±2.4a	2.1±0.5a	12.4±1.9a	18.9±2.8a	31.3±4.5a
		**LGBS**	4.0±1.1a	9.6±1.5a	8.5±1.6b	0.3±0.1b	13.6±2.5ab	9.0±1.6b	22.7±4.1a
		**HSIN**	5.0±0.5a	12.7±1.3a	11.2±1.2ab	1.6±0.7ab	18.0±1.3b	13.1±1.8ab	31.1±2.5a
		**LSIN**	4.3±1.2a	4.1±0.5b	8.5±2.9b	1.7±0.5ab	8.4±1.3a	10.4±3.0b	18.9±3.0a
	**JA**	**HGBS**	4.7±1.0a	6.7±2.0a	20.1±2.3a	5.9±1.1a	11.5±1.8a	26.4±2.3a	38.0±3.7a
		**LGBS**	6.6±4.1a	9.0±1.9a	12.1±2.6a	2.6±0.8a	15.6±2.7a	15.0±5.2b	30.5±4.3a
		**HSIN**	6.2±0.7a	18.5±2.1b	21.8±2.6a	5.8±1.7a	24.7±2.0b	28.6±2.7a	53.3±4.7b
		**LSIN**	5.6±1.5a	4.5±1.3a	20.7±3.8a	3.6±0.6a	10.3±2.5a	24.9±4.3a	35.2±3.7a
	**SA**	**HGBS**	2.5±0.5a	6.3±0.6a	13.6±2.6a	3.0±0.7a	8.8±0.9a	17.2±1.8a	26.1±2.0ab
		**LGBS**	3.8±0.8a	8.2±0.5a	7.4±2.5a	0.7±0.2a	12.4±0.5b	8.6±2.5b	21.0±3.0a
		**HSIN**	3.4±0.6a	12.9±1.3b	13.6±2.0a	1.5±0.4a	16.5±1.8c	15.4±2.2a	31.8±3.0b
		**LSIN**	3.8±0.7a	2.2±0.5c	9.3±1.5a	4.8±1.8a	6.0±0.5ad	14.4±1.4ab	20.4±1.7a
	**CL**	**HGBS**	0.9±0.2a	1.8±0.1a	20.6±2.4a	3.7±1.0a	4.5±0.3a	24.7±2.7a	29.2±2.5a
		**LGBS**	2.4±0.4ab	1.6±0.3a	18.4±3.6a	5.6±1.8a	5.5±0.8a	24.4±4.5a	29.9±4.7a
		**HSIN**	2.0±0.4ab	2.8±0.4a	22.1±3.7a	8.8±1.7a	8.6±1.3a	31.3±4.9a	39.8±4.5a
		**LSIN**	4.3±0.9b	1.3±0.7a	8.8±4.6a	7.8±3.4a	7.8±2.4a	17.0±5.0a	24.8±3.9a
	**JAL**	**HGBS**	1.7±0.9a	3.6±0.9a	36.8±2.5a	7.0±0.8a	5.3±1.7a	44.1±2.3a	49.4±3.9a
		**LGBS**	1.6±0.4a	2.9±1.0a	26.4±3.6a	3.8±1.2a	4.5±1.4a	30.4±3.5a	34.9±3.0a
		**HSIN**	3.1±1.1a	3.4±1.3a	25.8±5.3a	4.6±0.9a	6.5±1.4a	30.5±5.2a	37.7±5.3a
		**LSIN**	0.9±0.2a	2.3±1.6a	26.3±5.8a	6.9±2.2a	3.2±1.7a	33.5±8.0a	36.7±9.6a
	**SAL**	**HGBS**	2.0±0.4a	3.3±1.1a	18.8±1.9a	3.6±0.9a	5.3±1.3a	22.6±2.1a	27.9±3.2a
		**LGBS**	3.5±0.5a	6.2±1.1b	18.8±3.7a	2.5±0.5a	9.8±1.3b	21.5±4.2a	31.3±5.3a

**HSIN**	2.1±0.7a	7.5±0.6b	25.1±2.7a	3.7±1.0a	10.3±1.1b	29.3±2.8a	39.6±3.7a
**LSIN**	1.4±0.3a	1.0±0.2a	16.3±2.4a	4.8±1.5a	2.4±0.3a	21.5±2.8a	23.9±3.0a

Table S9. Effect of JA, SA, CL, JAL, and SAL treatments on glucosinolate content in the different genotypes, 1, 3, and 9 days after treatment with JA and SA. Test statistic and *P*-values of ANOVA or Kruskall-Wallis test shown to compare differences in glucosinolate content (percentages, compared to the control within each genotype) among genotypes subject to the same treatment. Significant *P*-values ($P \leq 0.05$) are shown in bold type.

**Days after treat.**	**Gluc.**	**JA**	**SA**	**CL**	**JAL**	**SAL**
**1**	**AL**	3.30; ***P*=0.031**	5.13; ***P*=0.005**	**-**	**-**	**-**
**1**	**IN**	3.06; ***P*=0.041**	3.20; ***P*=0.035**	**-**	**-**	**-**
**1**	**TO**	4.08; ***P*=0.014**	6.67; ***P*≤0.001**	**-**	**-**	**-**
**1**	**GIB**	5.00; ***P*=0.005**	8.74; ***P*≤0.001**	**-**	**-**	**-**
**1**	**SIN**	9.18; ***P*=0.027**	4.08; *P*=0.252	**-**	**-**	**-**
**1**	**GBS**	2.63; *P*=0.065	2.04; *P*=0.126	**-**	**-**	**-**
**1**	**NEO**	8.81; ***P*≤0.001**	4.30; ***P*=0.011**	**-**	**-**	**-**
**3**	**AL**	1.52; *P*=0.226	0.35; *P*=0.787	1.52; *P*=0.251	1.12; *P*=0.370	1.52; *P*=0.249
**3**	**IN**	4.16; ***P*=0.013**	0.83; *P*=0.488	8.39; ***P*=0.002**	4.97; ***P*=0.013**	1.59; *P*=0.231
**3**	**TO**	11.82; ***P*≤0.001**	0.94; *P*=0.431	12.56; ***P*≤0.001**	7.32; ***P*=0.003**	3.07; *P*=0.058
**3**	**GIB**	0.97; *P*=0.418	1.00; *P*=0.405	2.23; *P*=0.126	1.16; *P*=0.354	1.46; *P*=0.264
**3**	**SIN**	1.40; *P*=0.261	0.09; *P*=0.967	1.74; *P*=0.202	0.58; *P*=0.639	0.93; *P*=0.449
**3**	**GBS**	0.73; *P*=0.542	0.57; *P*=0.638	10.04; ***P*≤0.001**	6.25; ***P*=0.005**	1.71; *P*=0.206
**3**	**NEO**	3.35; ***P*=0.030**	0.87; *P*=0.464	0.54; *P*=0.662	2.85; *P*=0.071	0.91; *P*=0.458
**9**	**AL**	0.84; *P*=0.491	2.63; *P*=0.086	4.22; ***P*=0.027**	0.12; *P*=0.947	4.89 ***P*=0.014**
**9**	**IN**	2.86; *P*=0.070	1.40; *P*=0.280	2.50; *P*=0.106	2.00; *P*=0.160	3.28; ***P*=0.050**
**9**	**TO**	3.39; ***P*=0.044**	1.26; *P*=0.321	1.14; *P*=0.368	1.70; *P*=0.212	1.80; *P*=0.190
**9**	**GIB**	0.32; *P*=0.811	0.40; *P*=0.756	5.95; ***P*=0.009**	0.64; *P*=0.601	8.65; ***P*=0.034**
**9**	**SIN**	1.80; *P*=0.188	5.19; ***P*=0.011**	0.96; *P*=0.443	0.55; *P*=0.659	4.02; ***P*=0.028**
**9**	**GBS**	3.23; *P*=0.051	0.76; *P*=0.534	2.06; *P*=0.155	1.29; *P*=0.315	3.17; *P*=0.055
**9**	**NEO**	4.11; ***P*=0.024**	1.94; *P*=0.164	4.36; ***P*=0.025**	4.14; ***P*=0.027**	7.99; ***P*=0.002**

Table S10. Significance of correlations between aliphatic (AL) and indolic (IN) glucosinolate in induced plants. Data used in the correlations included all glucosinolate data from the four plant genotypes (HGBS, LGBS, HSIN, and LSIN) for each of the treatments, 1, 3 and 9 days after JA and SA treatment (n=39-40) and 3 and 9 days after CL, JAL, and SAL treatments began (n=19-20). Significant *P*-values ($P \leq 0.05$) of one-tailed Spearman's rho correlation are shown in bold type.

	**Correlations between AL and IN glucosinolate content (µmol g^{-1} plant dry weight) Spearman's rho correlation coefficients and *P*-values**				
	JA	SA	CL	JAL	SAL
1 day	0.417; ***P*=0.004**	0.362; ***P*=0.011**	-	-	-
3 days	-0.105; *P*=0.262	0.367; ***P*=0.011**	-0.400; ***P*=0.045**	-0.301; *P*=0.099	-0.305; *P*=0.095
9 days	0.134; *P*=0.287	-0.096; *P*=0.343	-0.017; *P*=0.474	0.104; *P*=0.340	0.523; ***P*=0.011**

Table S11. Differences in larval weights after feeding on leaf discs of the different plant genotypes and treatments during 9 days (n=8-10). *P*-values from Mann-Whitney U tests. Significant *P*-values ($P \leq 0.05$) are shown in bold type.

HGBS	C	JA
SA	*P*=0.491	***P*=0.029**
JA	***P*=0.028**	-
LGBS	C	JA
SA	*P*=0.161	*P*=0.236
JA	*P*=0.815	-
HSIN	C	JA
SA	*P*=0.321	***P*=0.019**
JA	***P*=0.006**	-
LSIN	C	JA
SA	*P*=0.075	***P*=0.034**
JA	***P*=0.001**	-

Table S12. Comparison of the percentage of leaf discs with defoliation ≥50% as a result of larval feeding under control (C), jasmonic acid (JA), and salicylic acid (SA) treatments. Significant *P*-values ($P \leq 0.05$) of one-tailed two-sample tests of proportions are shown in bold type.

	Comparison	C	JA	SA	Test statistic and *P*-value
**HGBS**	**C vs. JA**	52.4%	36.5%	-	*z*=0.68; *P*=0.249
**HGBS**	**C vs. SA**	52.4%	-	55.3%	*z*=0.12; *P*=0.548
**HGBS**	**JA vs. SA**	-	36.5%	55.3%	*z*=0.78; *P*=0.218
**LGBS**	**C vs. JA**	41.1%	47.6%	-	*z*=0.27; *P*=0.394
**LGBS**	**C vs. SA**	41.1%	-	46.4%	*z*=0.21; *P*=0.415
**LGBS**	**JA vs. SA**	-	47.6%	46.4%	*z*=0.05; *P*=0.480
**HSIN**	**C vs. JA**	65.1%	21.4%	-	***z*=1.93; *P*=0.027**
**HSIN**	**C vs. SA**	65.1%	-	39.3%	*z*=0.83; *P*=0.204
**HSIN**	**JA vs. SA**	-	21.4%	39.3%	*z*=1.06; *P*=0.144
**LSIN**	**C vs. JA**	57.1%	35.7%	-	*z*=0.90; *P*=0.183
**LSIN**	**C vs. SA**	57.1%	-	55.7%	*z*=0.06; *P*=0.475
**LSIN**	**JA vs. SA**	-	35.7%	55.7%	*z*=0.84; *P*=0.199

Table S13. Significance of correlations between plant glucosinolate content and larval weight at the end of the experiment (A) and between plant glucosinolate content and percentage of leaf discs with defoliation ≥ 50% (B). Correlations are shown for glucosinolate content 3 days and 9 days after JA and SA treatment. Data used were the glucosinolate averages corresponding to each plant genotype (HGBS, LGBS, HSIN, and LSIN) and treatment (C, JA, and SA) (n=12). Three different classes of glucosinolates were distinguished, aliphatic (AL), indolic (IN), and total (TO). Significant *P*-values ($P \leq 0.05$) are shown in bold type.

A

Correlations between plant glucosinolate content and larval weight<br>Pearson´s correlation coefficients and *P*-values						
	**AL 3 days**	**IN 3 days**	**TO 3 days**	AL 9 days	IN 9 days	TO 9 days
Coefficient	0.270	-0.853	-0.853	-0.435	-0.839	-0.818
P-value	0.198	**≤0.001**	**≤0.001**	0.079	**≤0.001**	**0.001**

B

Correlations between plant glucosinolate content and percentage of leaf discs with defoliation ≥ 50%<br>Pearson´s correlation coefficients and *P*-values						
	**AL 3 days**	**IN 3 days**	**TO 3 days**	**AL 9 days**	IN 9 days	TO 9 days
Coefficient	0.213	-0.729	-0.735	-0.600	-0.644	-0.768
P-value	0.253	**0.004**	**0.003**	**0.019**	**0.012**	**0.002**
